# Identifying age-common and age-specific factors of *Plasmodium* infection in Nigerian children under five: Application of a cluster-aware multistage selection framework to the 2018 Nigeria Demographic and Health Survey

**DOI:** 10.1371/journal.pgph.0006693

**Published:** 2026-07-14

**Authors:** Woojae Choi, Yunhee Kang, Joonsup Yeom

**Affiliations:** 1 Department of Internal Medicine, Metrowest Medical Center, Framingham, Massachusetts, United States of America; 2 Department of Food and Nutrition, Seoul National University College of Human Ecology, Seoul, Republic of Korea; 3 Department of Internal Medicine, Yonsei University College of Medicine, Seoul, Republic of Korea; PLOS: Public Library of Science, UNITED STATES OF AMERICA

## Abstract

Despite developmental heterogeneity, children under five are often analyzed as a single group, obscuring age-related differences in *Plasmodium* infection. Using the 2018 Nigeria Demographic and Health Survey data, we analyzed 2,914 younger children (6–23.9 months) and 5,553 older children (24–59 months) to identify age-common and age-specific factors. We applied a Cluster-aware Multistage Selection (CMS) framework integrating penalized regression, interaction testing, and bidirectional selection while accounting for sampling weights, clustering, and stratification. *Plasmodium* prevalence was 37.3% in younger children and 49.4% in older children. Each 10-percentage-point increase in community-level livestock and agricultural land ownership was associated with 2.5% (PR = 1.025; 95% CI: 1.011–1.039) and 4.6% (PR = 1.046; 95% CI: 1.029–1.062) higher prevalence, respectively. Socioeconomic disadvantage was associated with higher prevalence. Children in the lowest wealth quintile had 80.7% higher prevalence than those in the richest quintile (PR = 1.807; 95% CI: 1.467–2.224), and children whose mothers had no formal education had 55.8% higher prevalence than those with higher education (PR = 1.558; 95% CI: 1.225–1.980). Severe maternal anemia was associated with 22.5% higher prevalence (PR = 1.225; 95% CI: 1.012–1.483). Stunting and household insecticide-treated net (ITN) ownership were associated with 8.0% (PR = 1.080; 95% CI: 1.024–1.139) and 10.4% (PR = 1.104; 95% CI: 1.017–1.198) higher prevalence, respectively. Conversely, sleeping under an ITN (PR = 0.916; 95% CI: 0.858–0.977), breastfeeding (PR = 0.845; 95% CI: 0.764–0.934), maternal internet use (PR = 0.638; 95% CI: 0.475–0.857), and overweight-for-height (PR = 0.761; 95% CI: 0.631–0.917) were associated with lower prevalence. Age-specific associations were observed only in younger children, including paternal lack of education (PR = 1.301; 95% CI: 1.025–1.652) and rural residence (PR = 1.462; 95% CI: 1.266–1.688). These findings support age-tailored *Plasmodium* prevention and highlight the utility of CMS for complex population data.

## Introduction

Malaria remains a major cause of morbidity and mortality in Sub-Saharan Africa, with Nigeria accounting for 27% of global cases and 31% of malaria deaths in 2022 [1]. Children under five experience a disproportionately high *Plasmodium* infection burden that leads to malaria, with substantial contributions to severe anemia, neurocognitive impairment, and years of life lost [2,3]. Although usually analyzed as a single age group, children under five are developmentally heterogeneous. Infants and toddlers under two years—the “first 1,000 days”—experience rapid growth, immune immaturity, and nutritional vulnerability, whereas children aged two to five years gain greater independence, mobility, and environmental exposure [4–7]. Understanding age-specific risk factors for *Plasmodium* infection is critical for optimizing the targeting and effectiveness of prevention strategies.

The Demographic and Health Survey (DHS) provides nationally representative *Plasmodium* biomarker data through stratified, cluster-based sampling, primarily targeting children under five due to their high *Plasmodium* infection burden [8]. Most DHS-based *Plasmodium* infection studies pool children under five or compare age groups only descriptively [9–12]. These analyses often include many covariates to improve explanatory power, but this may increase collinearity, overfitting, and confounding [9,10,12]. Although methods such as purposeful selection and least absolute shrinkage and selection operator (LASSO) can mitigate these issues, they are rarely implemented in ways that fully account for the DHS’s stratified, cluster-based design [13–18].

We aimed to identify age-common and age-specific factors of *Plasmodium* infection among younger (6–23.9 months) and older (24–59 months) children using the 2018 Nigeria DHS. To do so, we developed the Cluster-aware Multistage Selection (CMS) framework, a three-stage, survey-design–aligned approach that evaluates both main effects and age interactions. Guided by prior literature, we hypothesized that socioeconomic factors would show age-common associations, whereas nutritional, environmental, and caregiver-related factors might vary by age due to differences in developmental vulnerability and exposure patterns [5–7,12,19,20].

## Materials and methods

### Data sources

We used de-identified data from the 2018 Nigeria DHS, conducted from August 14 to December 29, 2018. The DHS employs stratified two-stage cluster sampling to select approximately 5,000–30,000 households per country [8,15]. Our analysis included 8,447 children aged 6–59 months with available *Plasmodium* test results, corresponding to the population systematically sampled for *Plasmodium* testing in the DHS. Data were obtained from the DHS Program website ([href:https://dhsprogram.com/data/]https://dhsprogram.com/data/).

### Outcome measure: Plasmodium infection

As the DHS does not measure clinical malaria, we used *Plasmodium* infection as the outcome. Children were classified as infected if either light microscopy or rapid diagnostic tests (RDT) was positive, reflecting the high sensitivity of combined testing in high-endemic settings [21]. Records with one test missing and the other negative were excluded to improve specificity.

### Independent covariates

**Fig 1:**
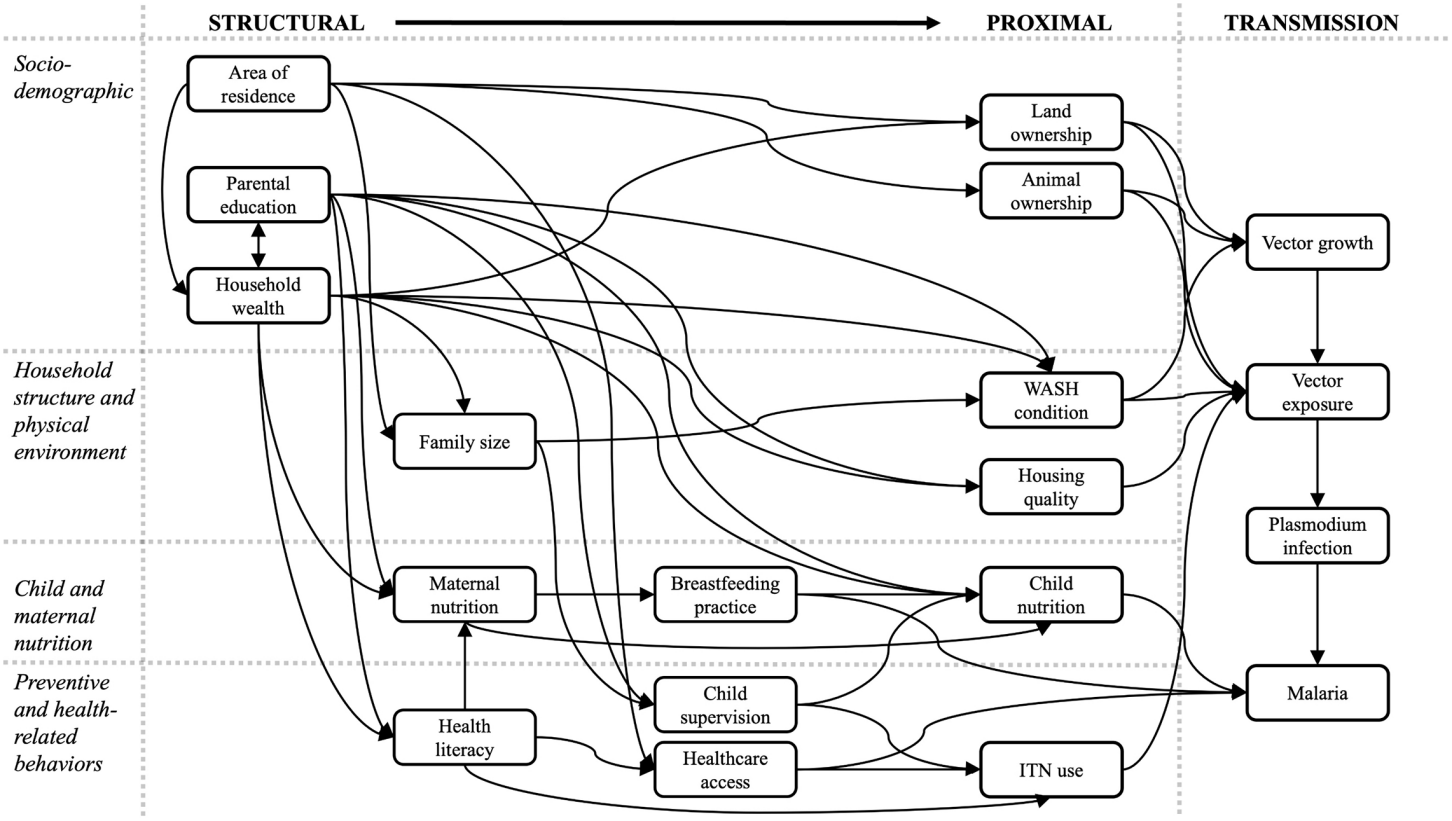
Directed acyclic graph (DAG) of factors associated with *Plasmodium* infection among children aged 6–59 months in Nigeria, 2018 Demographic and Health Survey. Conceptual DAG summarizing known and hypothesized pathways linking sociodemographic, environmental, nutritional, and behavioral factors to *Plasmodium* infection among children under five in Nigeria. The diagram integrates evidence from epidemiology, environmental health, child nutrition, and health-seeking behavior research and motivates identification of age-common and age-specific associations in the present analysis [5,9–11,14,19,20]. Abbreviations: DAG = directed acyclic graph; WASH = water, sanitation and hygiene; ITN = insecticide-treated net.

We selected 56 candidate covariates (excluding age) based on a conceptual directed acyclic graph (DAG; **Fig 1**), comprising 13 community-level and 43 individual-level variables. The DAG was specified to represent hypothesized antecedent factors; thus we did not depict potential reverse pathways from *Plasmodium* infection to covariates. We calculated community-level covariates as the proportion of children with each characteristic within each DHS primary sampling unit (PSU) and assigned these values to all individuals in that cluster. We used PSUs as the community unit because each corresponds to a census enumeration area, a well-defined geographic proxy for local communities in national surveys [8]. We extracted individual-level covariates from children’s DHS responses including 8 sociodemographic, 11 child and maternal nutrition, 14 household structure and physical-environment, and 10 preventive and health-related behavior variables. We excluded variables with extensive missingness (≥10%) or non-informative responses unless specified by DHS guidelines. We modeled age both as a continuous and a dichotomous variable (6–23.9 vs. 24–59 months). Covariate definitions are provided in **S1 Table**.

### Statistical analysis

We conducted all analyses in R (version 4.5.1) within RStudio. We implemented the Cluster-aware Multistage Selection (CMS) framework, a three-stage, survey-aligned variable-selection approach incorporating DHS sampling weights, PSU clustering, and stratification to identify age-common and age-specific factors. With no a priori thresholds, we tested multiple tuning combinations and selected the most accurate and parsimonious configuration.

#### 1. Step 1: survey-weighted LASSO regression covariate screening.

We fit a survey-weighted LASSO including all covariates and their interactions with the age-band indicator (6–23.9 vs. 24–59 months), leaving age and DAG-defined structural covariates unpenalized. Binary variables contributed one main and one interaction term; multi-category variables contributed these terms for each dummy indicator (a binary variable representing each response). We selected a parent covariate if any associated coefficient was non-zero at the chosen penalty parameter λ. To assess stability, we repeated LASSO across 200 stratified PSU-bootstrap replicates, advancing covariates selected above a tunable proportion threshold. Details on LASSO and bootstrapping are provided in **S1 Text**.

#### 2. Step 2: Age-common and age-specific candidate identification.

All retained covariates were entered into a survey-weighted generalized linear model including their main and interaction terms. We screened age-interaction blocks using Wald tests with tunable p-value thresholds and re-evaluated them across the same 200 stratified PSU-bootstrap replicates. We classified covariates passing the tunable threshold as candidate age-specific factors; others as age-common factors.

#### Step 3: Bidirectional selection for model enrichment.

We fitted a survey-weighted quasi-Poisson model with a log link including main-effect terms for all candidate age-common covariates and both main and interaction terms for candidate age-specific covariates. Backward elimination began from the full model. At each iteration, we marked terms not meeting any retention criterion for removal, and removed the term whose exclusion resulted in the smallest increase (or largest decrease) in the simplified quasi-likelihood information criterion (QICu; Pan 2001), ensuring that the terms contributing least to model fit were eliminated first [22].

Following backward elimination, we conducted a forward reassessment to re-evaluate previously excluded terms, including Step 1 main and interaction terms and Step 2 interaction terms of candidate age-common covariates. We added each candidate term individually, and among those satisfying any retention criterion, we reintroduced the term yielding the greatest improvement (or smallest decrease) in QICu. QICu ties in both directions were resolved by p-value, then alphabetically.

Retention criteria were: 1) statistical significance of Wald block tests; 2) improved or minimally worsened model fit based on dQICu; 3) evidence of confounding, defined as notable mean or maximum changes in other covariates’ PRs when a term was removed. Thresholds for each criterion (p-value, dQICu tolerance, PR-change cutoffs) were tunable. Main-effect components of retained interaction terms and all DAG-defined structural covariates were always preserved in the final model.

#### 3. Model diagnostics and selection of tuning parameters.

We evaluated all combinations of tunable thresholds across Steps 1–3. In step 1 we compared the minimum‐error and one–standard error rules for selecting the LASSO penalty λ, and bootstrap selection thresholds of 30% vs. 60%. In step 2 we evaluated age-interaction screening p-value cutoffs of 0.20, 0.15, and 0.10, alongside the same 30% and 60% bootstrap thresholds. Finally, in step 3 we assessed retention p-value thresholds of 0.15, 0.10, and 0.05 (always stricter than the paired Step 2 threshold), dQICu tolerances of −2, 0, and 2, and confounding thresholds based on relative mean and maximum PR changes of 10%/20% or 20%/40%.

For each configuration, we computed Pearson dispersion, QICu, calibration correlation, Rao–Scott F statistic, and total parameters (main and interaction terms counted separately). We used Brier scores obtained from cross-validation preserving DHS stratification and clustering as the primary ranking metric [23]. We grouped models into 0.1% bins above the minimum Brier score, with the k-th bin defined as $Brier_{min}(\text{1}+\text{0}.\text{0}\text{0}\text{1}\cdot k)$. We then ranked models by Brier‐score bins and, within bins, by parsimony based on the number of parameters.

We summarized retention patterns in a heatmap showing each covariate’s selection frequency and effect direction.

#### 4. Inference.

We used the top-ranked configuration to fit the final survey-weighted quasi-Poisson model. We assessed model fit using residual–fitted plots, and calibration by comparing survey-weighted observed prevalence with predicted probabilities across deciles.

For each covariate, the exponentiated main-effect coefficient represented the prevalence ratio (PR) among younger children. When an age interaction was present, the PR among older children was obtained by multiplying the exponentiated main-effect and interaction coefficients. Therefore, if the interaction coefficient was zero, the PR was identical across age groups, and the covariate was treated as age-common.

## Results

**Fig 2:**
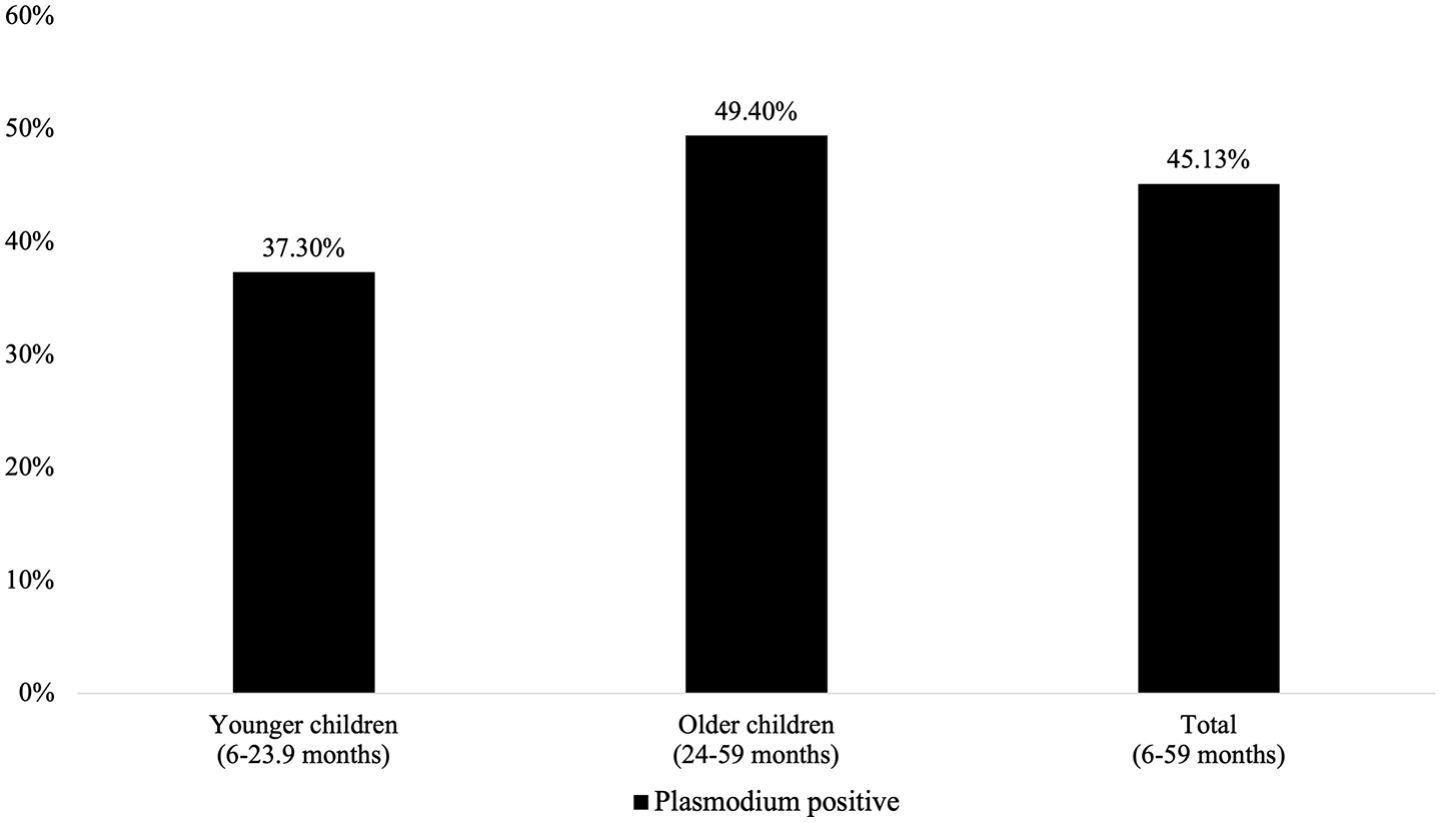
Prevalence of *Plasmodium* infection among younger (6–23.9 months) and older (24–59 months) children in Nigeria, 2018 Demographic and Health Survey. Weighted prevalence of *Plasmodium* infection among children aged 6–59 months, stratified by younger and older age groups. Children testing positive by either rapid diagnostic test or light microscopy were classified as *Plasmodium*-positive; those negative on both tests were classified as *Plasmodium*-negative. Estimates incorporate sampling weights, clustering, and geographic stratification.

We included a total of 2,914 younger children and 5,533 older children in the analysis. Among younger children, 37.3% tested positive for *Plasmodium* infection, compared with 49.4% of older children (**Fig 2**). Overall, 45.1% of children under five were positive (**S1 Fig**).

Weighted frequencies and proportions for individual-level covariates are presented in **Table 1A**, and median community-level proportions with their interquartile ranges (25th–75th percentiles) are shown in **Table 1B**. Full covariate definitions and recoding procedures are provided in **S1 Table**.

**Table 1:** A. Weighted distributions of individual-level covariates among younger (6–23.9 months) and older (24–59 months) children in Nigeria, 2018 Demographic and Health Survey. Weighted frequencies and proportions of each covariate in each age group are presented. Weights were calculated with clustering and geographical stratification according to the Demographic and Health Survey (DHS) methodologies. Each covariate was defined following the Demographic and Health Surveys (DHS) Standard Recode Manual for DHS-7 and the Guide to DHS Statistics, with additional refinements applied at the researchers’ discretion. Anthropometric indicators were coded according to World Health Organization definitions. Full details of covariate definitions and recoding procedures are provided in S1 Table. BMI = body mass index; ITN = insecticide-treated nets. 1B. Distributions of community-level covariates among younger (6–23.9 months) and older (24–59 months) children in Nigeria, 2018 Demographic and Health Survey. Distributions of community-level covariates across primary sampling units (PSU), each corresponding to a census enumeration area and serving as the operational community unit in this analysis. For each PSU, the proportion of children aged 6–59 months exhibiting each characteristic was calculated; median and interquartile ranges of these PSU-level proportions are reported.

Covariate name	Younger children N (%)	Older children N (%)
**Socioeconomic and demographic**		
**Female sex**	1403 (47.2%)	2703 (49.1%)
**Household wealth quintile**		
Richest	592 (19.9%)	999 (18.1%)
Richer	597 (20.1%)	1118 (20.3%)
Middle	614 (20.7%)	1126 (20.4%)
Poorer	563 (18.9%)	1118 (20.3%)
Poorest	605 (20.4%)	1146 (20.8%)
**Education level of the mother**		
Higher	288 (9.7%)	485 (8.8%)
Secondary	1097 (36.9%)	1800 (32.7%)
Primary	449 (15.1%)	942 (17.1%)
None	1138 (38.3%)	2279 (41.4%)
**Education level of the father**		
Higher	452 (15.2%)	783 (14.2%)
Secondary	1102 (37.1%)	1954 (35.5%)
Primary	411 (13.8%)	803 (14.6%)
None	1007 (33.9%)	1966 (35.7%)
**Occupation of the mother**		
Office work	1574 (53%)	3050 (55.4%)
Manual labor	124 (4.2%)	238 (4.3%)
None	1274 (42.9%)	2218 (40.3%)
**Occupation of the father**		
Office work	1208 (40.6%)	2191 (39.8%)
Manual labor	1524 (51.3%)	2900 (52.7%)
None	240 (8.1%)	415 (7.5%)
**Area of residence**		
North Central	398 (13.4%)	782 (14.2%)
North East	463 (15.6%)	827 (15%)
North West	871 (29.3%)	1699 (30.9%)
South South or South East	739 (24.9%)	1211 (22%)
South West	502 (16.9%)	988 (17.9%)
**Household lives in rural regions**	1634 (55%)	3111 (56.5%)
**Child & Maternal nutrition**		
**Fulfills minimum dietary diversity**	677 (22.8%)	0 (0%)
**Fulfills minimum meal frequency**	1230 (41.4%)	0 (0%)
**Fulfills minimum acceptable diet**	314 (10.6%)	0 (0%)
**Given iron supplement within 7 days**	548 (18.4%)	1038 (18.8%)
**Child given vitamin A supplement within 6 months**	1381 (46.5%)	2569 (46.6%)
**Anemia level of the mother**		
Normal	1279 (43%)	2278 (41.4%)
Mild	797 (26.8%)	1483 (26.9%)
Moderate	862 (29%)	1643 (29.8%)
Severe	34 (1.1%)	102 (1.8%)
**BMI status of the mother**		
Normal	1858 (62.5%)	3395 (61.7%)
Very thin	79 (2.7%)	148 (2.7%)
Thin	248 (8.3%)	349 (6.3%)
Overweight	527 (17.7%)	1055 (19.2%)
Obese	260 (8.8%)	560 (10.2%)
**Height-for-age**		
With stunting	981 (33%)	2349 (42.7%)
Normal	1991 (67%)	3157 (57.3%)
**Weight-for-height**		
With wasting	349 (11.8%)	229 (4.2%)
Normal	2543 (85.6%)	5166 (93.8%)
Overweight	80 (2.7%)	112 (2%)
**Weight-for-age**		
Underweight	682 (23%)	1248 (22.7%)
Normal	2261 (76.1%)	4238 (77%)
Overweight	28 (1%)	20 (0.4%)
**Currently breastfed**	1984 (66.8%)	90 (1.6%)
**Household structure and physical environment**		
**Number of household members**		
2-4 members	907 (30.5%)	1230 (22.3%)
5-7 members	1143 (38.5%)	2364 (42.9%)
8 or more members	922 (31%)	1912 (34.7%)
**Access to electricity**	1698 (57.1%)	3061 (55.6%)
**House has finished flooring material**	2117 (71.2%)	3889 (70.6%)
**2 or more rooms for sleeping**	2101 (70.7%)	4057 (73.7%)
**Cooks inside**	2012 (67.7%)	3725 (67.6%)
**Owns agricultural land**	1870 (62.9%)	3541 (64.3%)
**Owns livestock**	1438 (48.4%)	2955 (53.7%)
**Access to improved sanitation facility**	1649 (55.5%)	2875 (52.2%)
**Shares toilet with others**	896 (30.1%)	1661 (30.2%)
**Place of handwashing**		
At home: fixed	736 (24.8%)	1398 (25.4%)
At home: mobile	1673 (56.3%)	3092 (56.1%)
Not at home	563 (18.9%)	1016 (18.5%)
**Has soap and water when washing hands**	884 (29.8%)	1618 (29.4%)
**Ash, mud, sand present at handwashing place**	30 (1%)	60 (1.1%)
**Access to improved water source**	1865 (62.7%)	3466 (62.9%)
**Owns water source**	861 (29%)	1530 (27.8%)
**Prevention and health-related behaviors**		
**Household owns ITN**	2077 (69.9%)	3789 (68.8%)
**Household owns more than 1 ITN per 2 members**	566 (19%)	1042 (18.9%)
**Slept under an ITN last night**	1631 (54.9%)	2754 (50%)
**Mother uses the internet at least once a week**	281 (9.5%)	455 (8.3%)
**Mother exposed to mass media at least once a week**	1278 (43%)	2328 (42.3%)
**Visited health facility in the last 12 months**	1700 (57.2%)	2856 (51.9%)
**Mother had problems in accessing healthcare**	1578 (53.1%)	2918 (53%)
**Appropriate disposal of children's stool**	1650 (55.5%)	1269 (23%)
**Mother received drugs for intestinal parasites during pregnancy**	513 (17.3%)	459 (8.3%)
**Place of delivery**		
Own or other's home	1619 (54.5%)	3103 (56.3%)
Government facility	844 (28.4%)	1570 (28.5%)
Private facility	510 (17.1%)	833 (15.1%)
1B		
**Covariate**	**Median proportion** **(25th-75th percentile)**	
**Socioeconomic and demographic**		
**Household is poor or poorer**	20.0% (0.0%–80.0%)	
**Mother received primary education or higher**	88.9% (28.6%–100.0%)	
**Father received primary education or higher**	80.0% (50.0%–100.0%)	
**Mother is employed**	60.0% (33.3%–90.0%)	
**Father is employed**	100.0% (85.7%–100.0%)	
**Household structure and physical environment**		
**Household owns livestock**	50.0% (0.0%–83.3%)	
**Household owns agricultural land**	75.0% (28.6%–100.0%)	
**House has finished flooring material**	100.0% (50.0%–100.0%)	
**Access to electricity**	71.4% (0.0%–100.0%)	
**Access to improved sanitation facility**	57.1% (0.0%–100.0%)	
**Access to improved water source**	75.0% (33.3%–100.0%)	
**Prevention and health-related behaviors**		
**Mother exposed to mass media at least once a week**	40.0% (9.1%–75.0%)	
**Household owns ITN**	72.7% (42.9%–100.0%)	

A total of 144 tuning-parameter combinations were evaluated (**S2 Table**). Covariate-selection patterns were highly stable across models, with consistent retention of significant covariates, no reversals in effect direction, and non-significant covariates appearing mainly in lower-ranked models, indicating robustness of the selection procedure (**S2 Fig**). The highest-ranking model used the minimum-error rule for λ in Step 1, a stratified PSU-level bootstrap threshold of 30% in Steps 1 and 2, and a Step 2 screening threshold of p < 0.20. In Step 3, covariates were retained if they met at least one of the following: p < 0.05, model improvement by dQICu tolerance ≤2, or confounding PR changes ≥20% (mean) and ≥40% (maximum) among all other covariates. This model showed moderate overdispersion (Pearson dispersion = 3.75), no systematic residual–fitted patterns, and excellent calibration (calibration coefficient = 0.989), with close agreement between observed and predicted prevalence across risk deciles (**S3 Fig**). It was also the second-most parsimonious model, with 46 parameters.

Using these tuning parameters, 56 covariates entered Step 1, of which 35 were retained (**S3 Table**). In Step 2, 5 covariates were classified as age-common and 30 as candidate age-specific factors. After bidirectional selection in Step 3, 21 covariates remained, of which 17 were age-common and 4 were age-specific. All retained variables were selected during backward elimination, and none were reintroduced during forward reassessment.

PRs from the final model are shown in **Table 2**. Community-level PRs reflect prevalence changes per 10-percentage-point increase in PSU-level exposure, and individual-level PRs compare children with versus without the characteristic.

**Table 2:** Prevalence ratios for age-common and age-specific factors associated with Plasmodium infection among children aged 6–59 months in Nigeria. Prevalence ratios (PRs) and 95% confidence intervals (CIs) estimated from a survey-weighted generalized linear model using a three-stage, survey-aligned variable-selection framework to identify age-common and age-specific associations. Younger children were aged 6–23.9 months and older children 24–59 months. Age-common factors exhibit consistent associations across both age groups, whereas age-specific factors differ between them. Covariates associated with higher prevalence are shown in red; those associated with lower prevalence are shown in blue. Abbreviations: PR = prevalence ratio; CI = confidence interval; PSU = primary sampling unit; BMI = body mass index; ITN = insecticide-treated net.

Covariate name	Age pattern	Younger children PR (95% CI)	Older children PR (95% CI)
***Community-level factors (per 10-percentage-point increase)***			
**Socioeconomic and demographic**			
**Household is poor or poorer**	Age-common	1.013 (1.000–1.026)	
**Mother received primary education or higher**	Age-common	1.000 (0.982–1.018)	
**Household structure and physical environment**			
**Household owns livestock**	Age-common	**1.025 (1.011–1.039)**	
**Household owns agricultural land**	Age-common	**1.046 (1.029–1.062)**	
***Individual-level factors***			
**Socioeconomic and demographic**			
**Household wealth quintile**	Age-common		
Richest		Reference	
Richer		**1.668 (1.384–2.012)**	
Middle		**1.833 (1.515–2.218)**	
Poorer		**1.753 (1.432–2.147)**	
Poorest		**1.807 (1.467–2.224)**	
**Education level of the mother**	Age-common		
Higher		Reference	
Secondary		**1.303 (1.045–1.624)**	
Primary		**1.544 (1.228–1.941)**	
None		**1.558 (1.225–1.980)**	
**Education level of the father**	Age-specific		
Higher		Reference	Reference
Secondary		1.095 (0.858–1.397)	0.944 (0.567–1.570)
Primary		1.173 (0.895–1.537)	0.972 (0.557–1.696)
None		**1.301 (1.025–1.652)**	0.966 (0.592–1.575)
**Area of residence**	Age-common		
North Central		Reference	
North East		**0.763 (0.677–0.860)**	
North West		0.967 (0.868–1.077)	
South South or South East		1.078 (0.953–1.219)	
South West		**1.559 (1.367–1.778)**	
**Household lives in rural regions**	Age-specific	**1.462 (1.266–1.688)**	1.263 (0.950–1.679)
**Child & Maternal nutrition**			
**Given iron supplement within 7 days**	Age-specific	0.879 (0.757–1.021)	1.078 (0.795–1.462)
**Child given vitamin A supplement within 6 months**	Age-common	**0.936 (0.880–0.995)**	
**Anemia level of the mother**	Age-common		
Normal		Reference	
Mild		**1.132 (1.060–1.208)**	
Moderate		**1.144 (1.077–1.215)**	
Severe		**1.225 (1.012–1.483)**	
**BMI status of the mother**	Age-common		
Normal		Reference	
Very thin		0.975 (0.869–1.095)	
Thin		0.956 (0.874–1.046)	
Overweight		**0.896 (0.825–0.974)**	
Obese		**0.788 (0.669–0.928)**	
**Height-for-age**	Age-common		
With stunting		**1.080 (1.024–1.139)**	
Normal		Reference	
**Weight-for-height**	Age-common		
With wasting		1.047 (0.953–1.150)	
Normal		Reference	
Overweight		**0.761 (0.631–0.917)**	
**Currently breastfed**	Age-common	**0.845 (0.764–0.934)**	
**Household structure and physical environment**			
**Place of handwashing**	Age-common		
**At home: fixed**		Reference	
**At home: mobile**		1.005 (0.931–1.084)	
**Not at home**		1.091 (0.996–1.196)	
**Prevention and health-related behaviors**			
**Household owns ITN**	Age-common	**1.104 (1.017–1.198)**	
**Slept under an ITN last night**	Age-common	**0.916 (0.858–0.977)**	
**Mother uses the internet at least once a week**	Age-common	**0.638 (0.475–0.857)**	
**Appropriate disposal of children's stool**	Age-specific	0.942 (0.850–1.044)	1.073 (0.866–1.330)

Sixteen covariates had at least one response category significantly associated with *Plasmodium* infection (**Table 2**). Two community-level covariates showed age-common effects. A 10-percentage-point increase in community-level household livestock ownership was associated with a 2.5% increase in prevalence (PR = 1.025; 95% CI: 1.011–1.039), and a similar increase in community-level agricultural land ownership was associated with a 4.6% increase (PR = 1.046; 95% CI: 1.029–1.062).

Of the 14 individual-level covariates, 12 exhibited age-common effects and 2 were age-specific. Among age-common covariates associated with increased *Plasmodium* prevalence, the largest increases were observed for socioeconomic disadvantage. Children in any wealth quintile below the richest had prevalence increases of 66.8–80.7% (PR = 1.668–1.807; all p < 0.05), followed by children whose mothers had less than higher education, who had increases of 30.3–55.8% (PR = 1.303–1.558; all p < 0.05). Maternal anemia was associated with modest increases of 13.2–22.5% (PR = 1.132–1.225). Smaller increases were observed for household ITN ownership (PR = 1.104; 95% CI: 1.017–1.198) and child stunting (PR = 1.080; 95% CI: 1.024–1.139).

Several age-common covariates were associated with decreased *Plasmodium* prevalence. Children whose mothers used the internet at least once a week showed the largest reduction, with 36.2% lower prevalence (PR = 0.638; 95% CI: 0.475–0.857), followed by children who were overweight-for-height, with a 23.9% reduction (PR = 0.761; 95% CI: 0.631–0.917), and by children of mothers with obesity, who had a 21.2% reduction (PR = 0.788; 95% CI: 0.669–0.928). More modest reductions were observed for current breastfeeding with 15.5% lower prevalence (PR = 0.845; 95% CI: 0.764–0.934), maternal overweight with a 10.4% reduction (PR = 0.896; 95% CI: 0.825–0.974), sleeping under an ITN with an 8.4% reduction (PR = 0.916; 95% CI: 0.858–0.977), and recent vitamin A supplementation with a 6.4% reduction (PR = 0.936; 95% CI: 0.880–0.995).

Geographic region showed substantial variability in *Plasmodium* prevalence. Compared with the North Central region, prevalence was 23.7% lower in the North East (PR = 0.763; 95% CI: 0.667–0.860) and 55.9% higher in the South West (PR = 1.559; 95% CI: 1.367–1.778).

Two covariates demonstrated age-specific effects. Among younger children, having a father with no education versus higher education increased prevalence by 30.1% (PR = 1.301; 95% CI: 1.025–1.652), but no significant association was observed among older children (PR = 0.966; 95% CI: 0.592-1.575). Younger children living in rural areas also had markedly higher prevalence, with a 46.2% increase (PR = 1.462; 95% CI: 1.266–1.688), again with no significant association among older children (PR = 1.263; 95% CI: 0.950-1.679).

## Discussion

In this nationally representative analysis of Nigerian children under five, we applied the Cluster-aware Multistage Selection (CMS) framework to identify age-common and age-specific factors of *Plasmodium* infection while accounting for the stratified and clustered design of the Demographic and Health Survey. Using CMS, we reaffirmed established socioeconomic, nutritional, ecological, and behavioral determinants and revealed age-dependent heterogeneity obscured in pooled under-five analyses.

We found that community-level ecological factors of livestock and agricultural land ownership were consistently associated with higher *Plasmodium* prevalence. We observed geographic heterogeneity, with lower prevalence in the North East and higher prevalence in the South West relative to the North Central region.

Among age-common individual-level factors, we found that socioeconomic disadvantage showed the strongest associations, with clear gradients by household wealth and maternal education. Nutritional factors showed smaller but consistent associations, with stunting associated with higher prevalence and overweight or obesity, vitamin A supplementation, and breastfeeding associated with lower prevalence. Maternal anemia was associated with higher prevalence, whereas maternal overweight or obesity was associated with lower prevalence. Prevention-related behaviors showed divergent patterns, with ITN ownership associated with higher prevalence but child-level ITN use associated with lower prevalence. Maternal internet use showed the strongest protective association with *Plasmodium* prevalence.

We identified lack of paternal education and rural residence as age-specific factors; each associated with higher prevalence only among younger children.

Finally, we demonstrated the utility of CMS for high-dimensional population surveys. CMS enabled robust variable selection while accounting for complex survey design, yielding stable and interpretable models for population-level inference.

The associations between community-level livestock ownership, agricultural land use, and higher *Plasmodium* prevalence align with prior evidence that *Plasmodium* infection transmission is shaped primarily by shared ecological environments rather than individual household characteristics. Agricultural activity and livestock presence serve as proxies for vector-favorable landscapes that increase exposure risk at the community level [24–26].

We identified geographic heterogeneity that aligns with prior evidence of regional variation in *Plasmodium* infection burden across Nigeria [27]. Although both the North East and South West are ecologically suitable for *Anopheles* vectors, the lower prevalence observed in the North East after socioeconomic adjustment likely reflects differences in population density and *Plasmodium* control coverage, including seasonal malaria chemoprevention (SMC) [26,28–30]. In contrast, persistently higher prevalence in the highly urbanized South West is consistent with evidence that urbanization does not uniformly confer protection, as unplanned growth, poor drainage, and stagnant water can sustain *Anopheles* breeding even in nominally urban settings [28].

Strong and graded associations between socioeconomic disadvantage and *Plasmodium* prevalence are well documented [10–12,31,32]. Prior studies suggest that household wealth and maternal education reflect disparities in housing quality, preventive access, health literacy, and care-seeking linked to *Plasmodium* susceptibility [1,19,31,33,34]. Evidence also supports bidirectional causation, whereby *Plasmodium* infection may reinforce socioeconomic disadvantage through financial burden [35].

The associations observed for nutritional and maternal health factors align with established links between early-life nutrition, immune competence, and *Plasmodium* infection susceptibility. Chronic undernutrition may impair immune responses and parasite clearance, while malaria from *Plasmodium* infection may contribute to growth faltering, suggesting a bidirectional association with *Plasmodium* infection [36–38]. Breastfeeding and vitamin A supplementation are known to support immune function and have been associated with reduced *Plasmodium* infection [39,40].

Divergent associations for prevention-related behaviors warrant careful interpretation. Household ITN ownership was associated with higher prevalence, likely reflecting residual confounding and programmatic targeting of ITNs to higher-risk households in endemic areas, rather than a causal effect. In contrast, child-level ITN use was associated with lower prevalence, consistent with established protective efficacy [1,11,41]. This discrepancy highlights a measurement distinction between access and utilization, as ownership does not ensure consistent or correct use [42]. Additionally, reverse causality may contribute, whereby households experiencing prior malaria episodes are more likely to acquire or retain ITNs. Together, these findings emphasize that effective utilization, rather than access alone, is critical for reducing *Plasmodium* infection risk. Protective association for maternal internet use may reflect improved access to health information, as suggested by prior regional studies from Nigeria [43].

We identified age-specific associations for paternal education and rural residence, contrasting with prior pooled analyses that reported inconsistent or uniformly elevated risks, suggesting masked age-related heterogeneity [24,27,44–47]. Early childhood is a period of heightened caregiver dependence, when caregiver characteristics may disproportionately influence child health [34,43,46]. Therefore, paternal education may be more consequential in early childhood, before caregiving roles shift toward mothers as children age, and younger children in rural settings may experience greater exposure through caregiver proximity; however, these pathways could not be evaluated using DHS data.

Our findings among children under five align with *Plasmodium* infection patterns reported in older children and adults. Unlike children under five, there is no DHS-equivalent standardized survey that reliably estimates *Plasmodium* prevalence in older age groups in sub-Saharan Africa [48]. Global Burden of Disease data analyses across sub-Saharan Africa demonstrated lower *Plasmodium* prevalence among school-aged children (6–12 years; 17.6%) compared to adolescents and adults (≥12 years; 36.5%), mimicking our finding of higher prevalence among older children (49.4%) compared with younger children (37.3%) [48]. Across age groups, evidence suggests a repeating age-related gradient in *Plasmodium* prevalence, with lower prevalence among younger children and primary school–aged children and higher prevalence among older children under five and adolescents, indicating that ages two and twelve may represent key transition points in mobility, exposure, and caregiving intensity. Age-common factors identified in our study, including lower socioeconomic status, limited education, and inconsistent ITN use, were also significant determinants in older age groups [48–52].

Notably, some studies have found that rural residence has not been consistently associated with *Plasmodium* infection beyond age five once specific environmental and behavioral exposures are considered [49,53]. Instead, proximate factors such as household farming activity, stagnant water, proximity to rivers, and vegetation near households appear to drive infection risk, suggesting that the age-specific association observed among younger children in our study may also reflect the impact of age-dependent behaviors or environmental exposures rather than rural residence itself [49,54].

Methodologically, many DHS-based multivariable studies are limited by collinearity, inadequate model validation, and incomplete appreciation of the survey design, compromising stable selection and population-level inference [13,15,16,18]. These challenges likely contribute to heterogeneous findings and underscore the utility of CMS as a rigorous yet flexible approach.

Our study has several limitations. Because the 2024 DHS discontinued *Plasmodium* infection testing and the 2021 Malaria Indicator Survey did not collect covariates as comprehensive as those in the DHS, we relied on the 2018 DHS data, which may limit the timeliness of our findings. The cross-sectional DHS design supports association analyses but precludes assessment of transmission seasonality and evaluation of malaria control programs, including seasonal malaria chemoprevention. DHS *Plasmodium* infection testing is restricted to children under five, which precludes assessment of older children and limits inference across the full pediatric age spectrum. Outcome misclassification is possible due to persistent RDT positivity. Residual confounding from unmeasured environmental or behavioral factors may also influence observed associations. CMS relies on researcher-defined, data-informed tuning parameters, which may introduce selection uncertainty, and age was modeled using pre-specified categories rather than flexible nonlinear functions, potentially obscuring more complex age-related patterns. While CMS incorporated stability thresholds and survey-aligned validation to mitigate overfitting, post-selection bias cannot be fully eliminated.

## Conclusion

Using the 2018 Nigeria DHS, we applied the Cluster-aware Multistage Selection (CMS) framework to identify age-common and age-specific determinants of *Plasmodium* infection among children under five. Higher *Plasmodium* prevalence among younger children in rural and socioeconomically disadvantaged households highlights priority populations for age-targeted *Plasmodium* control, including household-level prevention and caregiver-focused interventions. CMS provides a rigorous, reproducible approach for inference in clustered, multilevel data and is broadly applicable to complex population-based studies such as DHS. Public health interventions should account for age heterogeneity within under-five populations, and future studies should investigate underlying immunological, behavioral, and socioeconomic mechanisms while validating CMS across settings.

## Supporting information

S1 Table Full list and definitions of covariates evaluated in the Cluster-aware Multistage Selection to identify age-common and age-specific factors associated with Plasmodium infection among children under 5 years in Nigeria, based on the 2018 Nigeria Demographic and Health Survey. Raw covariates were merged, filtered, or recoded as needed to create more interpretable, generalizable, and epidemiologically meaningful variables. The Demographic and Health Survey (DHS) Standard Recode Manual and the Guide to DHS Statistics (DHS-7) were consulted in constructing these covariates.
(XLSX)

S2 Table Diagnostic assessments for models used to tune threshold parameters in the Cluster-aware Multistage Selection identifying age-common and age-specific factors associated with Plasmodium infection among children under five in Nigeria, 2018 Nigeria Demographic and Health Survey. Tuning parameters included the method (minimum-error or 1-standard-error) used to determine the penalty λ term for least absolute shrinkage and selection operator regression in Step 1; the proportion of bootstrap trials required for a covariate to pass Step 1; the p-value cutoff for identifying candidate age-specific covariates in Step 2; the p-value cutoff for selecting significant covariates in Step 3; the cutoff of modified quasi-likelihood information criterion improvement for identifying model-improving covariates in Step 3; and the cutoff of relative prevalence ratio changes for identifying potential confounding covariates in Step 3. For each model, we report the Pearson dispersion statistic, the modified quasi-likelihood information criterion, the calibration correlation coefficient, the Rao–Scott adjusted global model test, the cross-validated Brier score and bins grouped by 0.1% increments of the best score, and the number of estimated parameters. Models were ranked first by their Brier score bins and, within ties, by parsimony. Selected model is highlighted in black. The tuning parameters from the top-ranked model were selected. Abbreviations: F = Rao-Scott F statistic; λ = penalty term used for least absolute shrinkage and selection operator regression; dQIC = difference in the simplified quasi-likelihood information criterion; dPR = change in prevalence ratio; φ = Pearson dispersion statistic; QICu = simplified quasi-likelihood information criterion; r = calibration correlation coefficient.
(XLSX)

S3 Table Step-by-step results of the Cluster-aware Multistage Selection identifying age-common and age-specific factors associated with Plasmodium infection among children under five in Nigeria, 2018 Nigeria Demographic and Health Survey. Variable selection results from the three-stage covariate screening procedure. Community-level factors were defined as primary sampling unit level proportions of individuals exhibiting each covariate, and individual-level factors were measured directly for each child. In Step 1, survey-weighted least absolute shrinkage and selection operator with stratified primary sampling unit bootstrapping retained covariates selected in ≥60% of bootstrap replicates. In Step 2, generalized linear models (GLMs) with bootstrapping identified candidate age-varying covariates when age-interaction terms were significant in ≥60% of replicates; remaining covariates were treated as candidate age-common covariates. In Step 3, backward elimination with forward reassessment retained covariates that showed significant associations with Plasmodium infection, improved model fit by modified quasi-information criterion changes, or acted as confounders based on changes in mean or maximum prevalence ratios. Refer to S1 Text for details. The final age pattern (“age-common” or “age-specific”) and retention status are reported for each covariate. Abbreviations: LASSO = Least Absolute Shrinkage and Selection Operator; GLM = Generalized Linear Model; DAG = Directed Acyclic Graph; BMI = Body Mass Index; ITN = Insecticide-Treated Net; QICu = simplified quasi-likelihood information criterion; dQIC = QICu of reduced model minus QICu of retained model; PR = prevalence ratio; dPR = Relative prevalence ratio change.
(XLSX)

S4 Table Covariate-level results from the final Cluster-aware Multistage Selection model identifying age-common and age-specific factors associated with Plasmodium infection among children under five in Nigeria, 2018 Nigeria Demographic and Health Survey. Community-level factors were defined as primary sampling unit level proportions of individuals exhibiting each covariate, and individual-level factors were measured directly for each child. Model statistics are reported for covariates retained in the final survey-weighted quasi-Poisson regression model with a log link following the multi-level, three-stage, survey-design-aware, bidirectional covariate selection procedure. Interaction terms between the age-bin indicator (24–59 months versus 6–23.9 months) and each covariate X are presented as Age × X. Abbreviations: CI = confidence interval; ITN = insecticide-treated nets.
(XLSX)

S1 Fig Prevalence of Plasmodium infection among younger (6–23.9 months) and older (24–59 months) children in Nigeria, 2018 Demographic and Health Survey. Weighted prevalence of Plasmodium infection among children aged 6–59 months, stratified by younger and older age groups. Children testing positive by either rapid diagnostic test or light microscopy were classified as malaria-positive; those negative on both tests were classified as malaria-negative. Estimates incorporate sampling weights, clustering, and geographic stratification.
(TIF)

S2 Fig Covariate retention across 144 tuning configurations in the Cluster-aware Multistage Selection identifying age-common and age-specific factors associated with Plasmodium infection among children under five in Nigeria, 2018 Nigeria Demographic and Health Survey. The Cluster-aware Multistage Selection framework included several tunable components. Step 1 varied the LASSO penalty-rule for λ (minimum-error vs 1-standard-error). Steps 1–2 varied the stratified PSU-level bootstrap selection threshold (30% vs 60%) and Step 2 used p-value cutoffs of 0.20, 0.15, or 0.05. Step 3 applied stricter p-value thresholds (0.15, 0.10, 0.05), simplified quasi-likelihood information criterion or QICu of reduced model minus retained model tolerances of (−2, 0, or +2), and confounding thresholds based on relative mean/maximum PR changes (20%/40% or 10%/20%). These combinations yielded 144 unique tuning configurations. Models were ranked by Brier-score bins and, within bins, by parsimony. In the plot, models are ordered left to right from highest to lowest ranking. For each model, covariates are shown as retained (X) or excluded; if retained, associations are colored as significant increasing (red), significant decreasing (blue), or non-significant (grey). Results are presented separately for younger (6–23.9 months) and older (24–59 months) children, with age-common and age-specific associations distinguished by shading intensity. Abbreviations: BMI = body mass index; ITN = insecticide-treated nets.
(TIF)

S3 Fig Residual and calibration plots for the final model selected using the Cluster-aware Multistage Selection to identify age-common and age-specific factors associated with Plasmodium infection among children under five in Nigeria, 2018 Nigeria Demographic and Health Survey. Model selected from the Cluster-aware Multistage Selection was evaluated using residual–fitted plots, and calibration was assessed by comparing survey-weighted observed prevalence with predicted probabilities across deciles of fitted probabilities.
(TIF)

S1 Text Statistical methods used in the Cluster-aware Multistage Selection to identify age-common and age-specific factors of Plasmodium infection among children under five in Nigeria, using the 2018 Nigeria Demographic and Health Survey. (PDF)

## References

[1] World Health Organization. World Malaria Report 2024. Geneva: World Health Organization; 2024.

[2] Trivedi S, Chakravarty A. Neurological complications of malaria. Current Neurology and Neuroscience Reports. 2022. doi: 10.1007/s11910-022-01214-6 PMC9192935 35699901

[3] Angell B, Sanuade O, Adetifa IMO, Okeke IN, Adamu AL, Aliyu MH. Population health outcomes in Nigeria compared with other west African countries, 1998–2019: a systematic analysis for the Global Burden of Disease Study. The Lancet. 2022;399(10330). doi: 10.1016/S0140-6736(21)02722-7 PMC8943279 35303469

[4] Ebrahim GJ. WHO child growth standards. growth velocity based on weight, length and head circumference. J Trop Pediatr. 2010;56(2). doi: 10.1093/tropej/fmp086

[5] Kihwele F, Gavana T, Makungu C, Msuya HM, Mlacha YP, Govella NJ, et al. Exploring activities and behaviours potentially increases school-age children’s vulnerability to malaria infections in south-eastern Tanzania. Malar J. 2023;22(1):293. doi: 10.1186/s12936-023-04703-2 37789435 PMC10548596

[6] Victora CG, Adair L, Fall C, Hallal PC, Martorell R, Richter L, et al. Maternal and child undernutrition: consequences for adult health and human capital. Lancet. 2008;371(9609):340–57. doi: 10.1016/S0140-6736(07)61692-4 18206223 PMC2258311

[7] Scott JA. The first 1000 days: a critical period of nutritional opportunity and vulnerability. Nutr Diet. 2020;77(3):295–7. doi: 10.1111/1747-0080.12617 32478460

[8] National Population Commission (NPC) N, ICF. Nigeria Demographic Health Survey 2018. Rockville, Maryland, USA: ICF; 2019.

[9] Nambiema A, Robert A, Yaya I. Prevalence and risk factors of anemia in children aged from 6 to 59 months in Togo: analysis from Togo demographic and health survey data, 2013-2014. BMC Public Health. 2019;19(1):215. doi: 10.1186/s12889-019-6547-1 30786883 PMC6383221

[10] Zgambo M, Mbakaya BC, Kalembo FW. Prevalence and factors associated with malaria parasitaemia in children under the age of five years in Malawi: a comparison study of the 2012 and 2014 Malaria Indicator Surveys (MISs). PLoS One. 2017;12(4):e0175537. doi: 10.1371/journal.pone.0175537 28399179 PMC5388476

[11] Sarfo JO, Amoadu M, Kordorwu PY, Adams AK, Gyan TB, Osman A-G, et al. Malaria amongst children under five in sub-Saharan Africa: a scoping review of prevalence, risk factors and preventive interventions. Eur J Med Res. 2023;28(1):80. doi: 10.1186/s40001-023-01046-1 36800986 PMC9936673

[12] Anjorin S, Okolie E, Yaya S. Malaria profile and socioeconomic predictors among under-five children: an analysis of 11 sub-Saharan African countries. Malar J. 2023;22(1):55. doi: 10.1186/s12936-023-04484-8 36788541 PMC9927033

[13] Bursac Z, Gauss CH, Williams DK, Hosmer DW. Purposeful selection of variables in logistic regression. Source Code Biol Med. 2008;3:17. doi: 10.1186/1751-0473-3-17 19087314 PMC2633005

[14] Lakew G, Tareke AA, Yigzaw ZA, Getachew D, Getachew E, Yirsaw AN. Individual and community level factors of malaria among under-five children in Kenya: based on the Kenya Demographic and Health Survey. PLoS One. 2025;20(10):e0335346. doi: 10.1371/journal.pone.0335346 41144485 PMC12558447

[15] Corsi DJ, Neuman M, Finlay JE, Subramanian SV. Demographic and health surveys: a profile. Int J Epidemiol. 2012;41(6):1602–13. doi: 10.1093/ije/dys184 23148108

[16] Friedman J, Hastie T, Tibshirani R. Regularization paths for generalized linear models via coordinate descent. J Stat Softw. 2010;33(1):1–22. doi: 10.18637/jss.v033.i01 20808728 PMC2929880

[17] Tibshirani R. Regression shrinkage and selection via the lasso. J Royal Statistical Society Series B: Methodological. 1996;58(1). doi: 10.1111/j.2517-6161.1996.tb02080.x

[18] Dey D, Haque MS, Islam MM, Aishi UI, Shammy SS, Mayen MSA, et al. The proper application of logistic regression model in complex survey data: a systematic review. BMC Med Res Methodol. 2025;25(1):15. doi: 10.1186/s12874-024-02454-5 39844030 PMC11752662

[19] Tusting LS, Bottomley C, Gibson H, Kleinschmidt I, Tatem AJ, Lindsay SW, et al. Housing improvements and malaria risk in sub-saharan africa: a multi-country analysis of survey data. PLoS Med. 2017;14(2):e1002234. doi: 10.1371/journal.pmed.1002234 28222094 PMC5319641

[20] Yang D, He Y, Wu B, Deng Y, Li M, Yang Q, et al. Drinking water and sanitation conditions are associated with the risk of malaria among children under five years old in sub-Saharan Africa: a logistic regression model analysis of national survey data. J Adv Res. 2019;21:1–13. doi: 10.1016/j.jare.2019.09.001 31641533 PMC6796660

[21] Mwenda MC, Fola AA, Ciubotariu II, Mulube C, Mambwe B, Kasaro R, et al. Performance evaluation of RDT, light microscopy, and PET-PCR for detecting Plasmodium falciparum malaria infections in the 2018 Zambia National Malaria Indicator Survey. Malar J. 2021;20(1):386. doi: 10.1186/s12936-021-03917-6 34583692 PMC8477358

[22] Pan W. Akaike’s information criterion in generalized estimating equations. Biometrics. 2001;57(1):120–5. doi: 10.1111/j.0006-341x.2001.00120.x 11252586

[23] Steyerberg EW, Vickers AJ, Cook NR, Gerds T, Gonen M, Obuchowski N, et al. Assessing the performance of prediction models. Epidemiology. 2010;21(1):128–38. doi: 10.1097/EDE.0b013e3181c30fb2 20010215 PMC3575184

[24] Janko MM, Irish SR, Reich BJ, Peterson M, Doctor SM, Mwandagalirwa MK, et al. The links between agriculture, Anopheles mosquitoes, and malaria risk in children younger than 5 years in the Democratic Republic of the Congo: a population-based, cross-sectional, spatial study. Lancet Planet Health. 2018;2(2):e74–82. doi: 10.1016/S2542-5196(18)30009-3 29457150 PMC5809714

[25] Arisco NJ, Rice BL, Tantely LM, Girod R, Emile GN, Randriamady HJ, et al. Variation in Anopheles distribution and predictors of malaria infection risk across regions of Madagascar. Malar J. 2020;19(1):348. doi: 10.1186/s12936-020-03423-1 32993669 PMC7526177

[26] Adeogun A, Babalola AS, Okoko OO, Oyeniyi T, Omotayo A, Izekor RT, et al. Spatial distribution and ecological niche modeling of geographical spread of Anopheles gambiae complex in Nigeria using real time data. Sci Rep. 2023;13(1):13679. doi: 10.1038/s41598-023-40929-5 37608210 PMC10444803

[27] Obasohan PE, Walters SJ, Jacques R, Khatab K. Individual and Contextual Factors Associated with Malaria among Children 6-59 Months in Nigeria: A Multilevel Mixed Effect Logistic Model Approach. Int J Environ Res Public Health. 2021;18(21):11234. doi: 10.3390/ijerph182111234 34769754 PMC8582856

[28] Merga H, Degefa T, Birhanu Z, Tadele A, Lee MC, Yan G. Urban malaria in sub-Saharan Africa: a scoping review of epidemiologic studies. Malar J. 2025;24(1):131. doi: 10.1186/s12936-025-05368-9 40253329 PMC12009534

[29] Ogbulafor N, Uhomoibhi P, Shekarau E, Nikau J, Okoronkwo C, Fanou NML, et al. Facilitators and barriers to seasonal malaria chemoprevention (SMC) uptake in Nigeria: a qualitative approach. Malar J. 2023;22(1):120. doi: 10.1186/s12936-023-04547-w 37041516 PMC10088202

[30] Ambe JP, Balogun ST, Waziri MB, Nglass IN, Saddiq A. Impacts of seasonal malaria chemoprevention on malaria burden among under five-year-old children in Borno State, Nigeria. J Trop Med. 2020;2020:9372457. doi: 10.1155/2020/9372457 32665781 PMC7349624

[31] Tusting LS, Willey B, Lucas H, Thompson J, Kafy HT, Smith R, et al. Socioeconomic development as an intervention against malaria: a systematic review and meta-analysis. Lancet. 2013;382(9896):963–72. doi: 10.1016/S0140-6736(13)60851-X 23790353

[32] Chilot D, Mondelaers A, Alem AZ, Asres MS, Yimer MA, Toni AT, et al. Pooled prevalence and risk factors of malaria among children aged 6-59 months in 13 sub-Saharan African countries: A multilevel analysis using recent malaria indicator surveys. PLoS One. 2023;18(5):e0285265. doi: 10.1371/journal.pone.0285265 37256889 PMC10231787

[33] Diema Konlan K, Amu H, Konlan KD, Japiong M. Awareness and Malaria Prevention Practices in a Rural Community in the Ho Municipality, Ghana. Interdiscip Perspect Infect Dis. 2019;2019:9365823. doi: 10.1155/2019/9365823 31239838 PMC6556342

[34] Adeleke TO, Akinlosotu MA, Samson TK, Adeleke OV, Ajala DE, Olasinde Y, et al. Malaria knowledge and preventive practices among caregivers of under-five children in Southwest Nigeria. Sci Rep. 2025;15(1):36821. doi: 10.1038/s41598-025-22713-9 41120734 PMC12540983

[35] Andrade MV, Noronha K, Diniz BPC, Guedes G, Carvalho LR, Silva VA, et al. The economic burden of malaria: a systematic review. Malar J. 2022;21(1):283. doi: 10.1186/s12936-022-04303-6 36199078 PMC9533489

[36] Gari T, Loha E, Deressa W, Solomon T, Lindtjørn B. Malaria increased the risk of stunting and wasting among young children in Ethiopia: results of a cohort study. PLoS One. 2018;13(1):e0190983. doi: 10.1371/journal.pone.0190983 29324840 PMC5764317

[37] Fillol F, Sarr JB, Boulanger D, Cisse B, Sokhna C, Riveau G, et al. Impact of child malnutrition on the specific anti-Plasmodium falciparum antibody response. Malar J. 2009;8:116. doi: 10.1186/1475-2875-8-116 19490641 PMC2700128

[38] Oldenburg CE, Guerin PJ, Berthé F, Grais RF, Isanaka S. Malaria and nutritional status among children with severe acute malnutrition in niger: a prospective cohort study. Clin Infect Dis. 2018;67(7):1027–34. doi: 10.1093/cid/ciy207 29522089 PMC6137121

[39] Serghides L, Kain KC. Mechanism of protection induced by vitamin A in falciparum malaria. Lancet. 2002;359(9315):1404–6. doi: 10.1016/S0140-6736(02)08360-5 11978340

[40] Brazeau NF, Tabala M, Kiketa L, Kayembe D, Chalachala JL, Kawende B, et al. Exclusive breastfeeding and clinical malaria risk in 6-month-old infants: a cross-sectional study from kinshasa, democratic republic of the Congo. Am J Trop Med Hyg. 2016;95(4):827–30. doi: 10.4269/ajtmh.16-0011 27549632 PMC5062781

[41] Nwaorgu OC. ITN ownership and usage among household members in Okposi and Umuoghana communities in Ezza North LGA, Ebonyi State, Nigeria. Int J Epidemiol. 2015;44(suppl_1):337. doi: 10.1093/ije/dyv096

[42] Eisele TP, Keating J, Littrell M, Larsen D, Macintyre K. Assessment of insecticide-treated bednet use among children and pregnant women across 15 countries using standardized national surveys. Am J Trop Med Hyg. 2009;80(2):209–14. doi: 10.4269/ajtmh.2009.80.209 19190215

[43] Igbinoba AO, Soola EO, Omojola O, Odukoya J, Adekeye O, Salau OP. Women’s mass media exposure and maternal health awareness in Ota, Nigeria. Cogent Social Sciences. 2020;6(1). doi: 10.1080/23311886.2020.1766260

[44] Okunlola O, Oloja S, Ebiwonjumi A, Oyeyemi O. Vegetation index and livestock practices as predictors of malaria transmission in Nigeria. Sci Rep. 2024;14(1):9565. doi: 10.1038/s41598-024-60385-z 38671079 PMC11053042

[45] Afoakwah C, Deng X, Onur I. Malaria infection among children under-five: the use of large-scale interventions in Ghana. BMC Public Health. 2018;18(1):536. doi: 10.1186/s12889-018-5428-3 29685120 PMC5913887

[46] Osborne A, Ahinkorah BO. The paternal influence on early childhood development in Africa: implications for child and adolescent mental health. Child Adolesc Psychiatry Ment Health. 2024;18(1):156. doi: 10.1186/s13034-024-00847-4 39643898 PMC11622458

[47] Bayode T, Siegmund A. Social determinants of malaria prevalence among children under five years: A cross-sectional analysis of Akure, Nigeria. Scientific African. 2022;16:e01196. doi: 10.1016/j.sciaf.2022.e01196

[48] Oshagbemi OA, Lopez-Romero P, Winnips C, Csermak KR, Su G, Aubrun E. Estimated distribution of malaria cases among children in sub-Saharan Africa by specified age categories using data from the Global Burden of Diseases 2019. Malar J. 2023;22(1):371. doi: 10.1186/s12936-023-04811-z 38053100 PMC10696666

[49] Ibrahim AO, Bello IS, Shabi OM, Omonijo AO, Ayodapo A, Afolabi BA. Malaria infection and its association with socio-demographics, preventive measures, and co-morbid ailments among adult febrile patients in rural Southwestern Nigeria: a cross-sectional study. SAGE Open Med. 2022;10:20503121221117853. doi: 10.1177/20503121221117853 36051785 PMC9424889

[50] Muhye A, Abate MA, Ayehu A, Dejen P. Asymptomatic plasmodium infection and predictors among schoolchildren in Bahir Dar Zuria District, Northwest Ethiopia. Malar J. 2025. doi: 10.1186/s12936-025-05695-x PMC12821173 41390626

[51] Ibrahim AO, Agbesanwa TA, Aremu SK, Bello IS, Elegbede OT, Gabriel-Alayode OE, et al. Malaria infection and its association with socio-demographics, long lasting insecticide nets usage and hematological parameters among adolescent patients in rural Southwestern Nigeria. PLoS One. 2023;18(7):e0287723. doi: 10.1371/journal.pone.0287723 37450497 PMC10348556

[52] Dawaki S, Al-Mekhlafi HM, Ithoi I, Ibrahim J, Atroosh WM, Abdulsalam AM, et al. Is Nigeria winning the battle against malaria? Prevalence, risk factors and KAP assessment among Hausa communities in Kano State. Malar J. 2016;15:351. doi: 10.1186/s12936-016-1394-3 27392040 PMC4938925

[53] Zerdo Z, Bastiaens H, Anthierens S, Massebo F, Masne M, Biresaw G, et al. Prevalence and associated risk factors of asymptomatic malaria and anaemia among school-aged children in Dara Mallo and Uba Debretsehay districts: results from baseline cluster randomized trial. Malar J. 2021;20(1):400. doi: 10.1186/s12936-021-03937-2 34645464 PMC8513194

[54] Onyiah AP, Ajayi IO, Dada-Adegbola HO, Adedokun BO, Balogun MS, Nguku PM, et al. Long-lasting insecticidal net use and asymptomatic malaria parasitaemia among household members of laboratory-confirmed malaria patients attending selected health facilities in Abuja, Nigeria, 2016: a cross-sectional survey. PLoS One. 2018;13(9):e0203686. doi: 10.1371/journal.pone.0203686 30212496 PMC6136754

