# Supplementary figures and images for: Identifying age-common and age-specific factors of Plasmodium infection in Nigerian children under five: Application of a cluster-aware multistage selection framework to the 2018 Nigeria Demographic and Health Survey
Source: PLOS Glob Public Health. 2026 Jul 14;6(7):e0006693. doi: 10.1371/journal.pgph.0006693 (PMC13367690; doi:10.1371/journal.pgph.0006693)

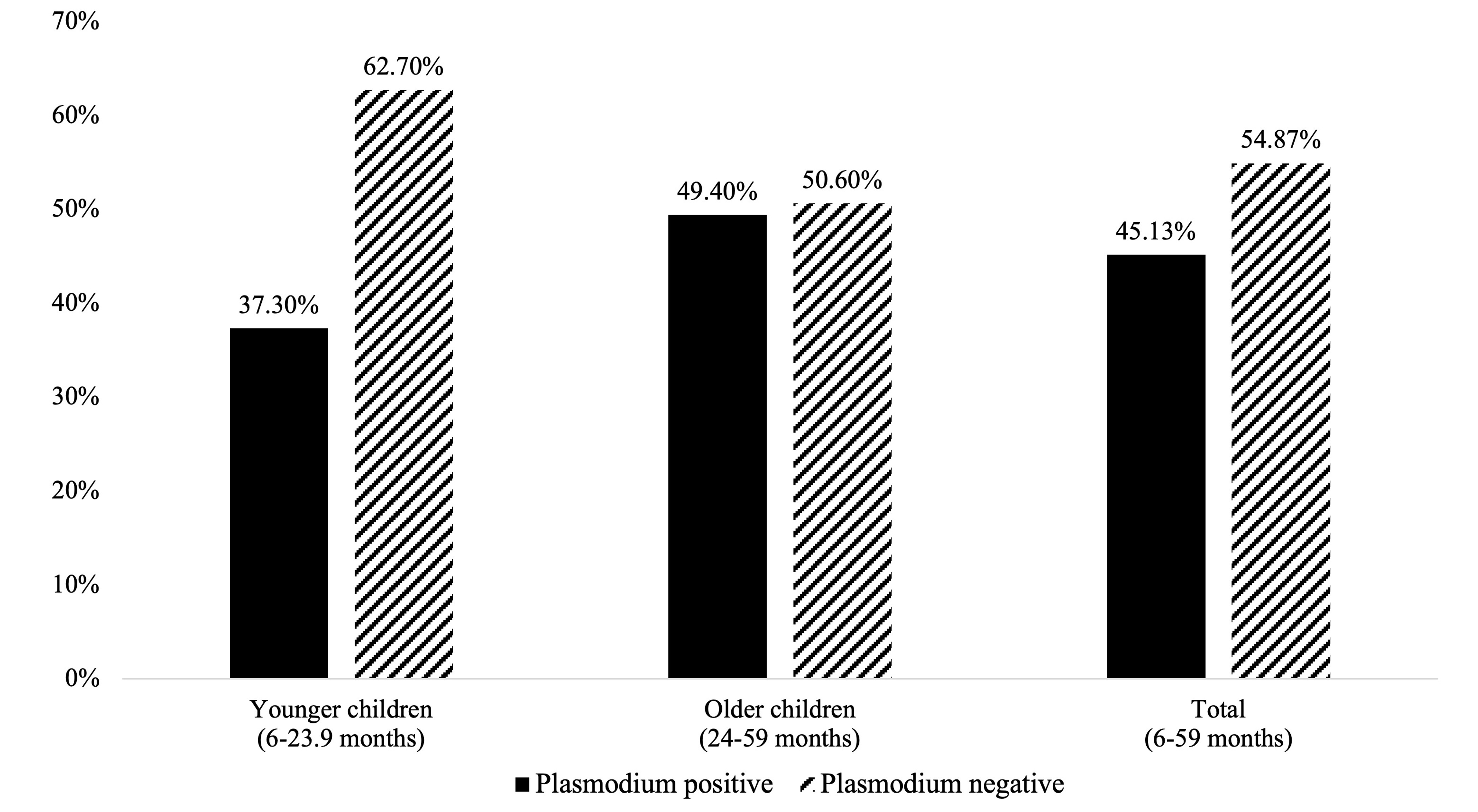

Supplement: S1 Fig — Weighted prevalence of Plasmodium infection among children aged 6–59 months, stratified by younger and older age groups. Children testing positive by either rapid diagnostic test or light microscopy were classified as malaria-positive; those negative on both tests were classified as malaria-negative. Estimates incorporate sampling weights, clustering, and geographic stratification. (TIF) [file pgph.0006693.s005.tif]
