# Supplementary material for: Identifying age-common and age-specific factors of Plasmodium infection in Nigerian children under five: Application of a cluster-aware multistage selection framework to the 2018 Nigeria Demographic and Health Survey
Source: PLOS Glob Public Health. 2026 Jul 14;6(7):e0006693. doi: 10.1371/journal.pgph.0006693 (PMC13367690; doi:10.1371/journal.pgph.0006693)
Supplement: S1 Text — (PDF) [file pgph.0006693.s008.pdf]

S1 Text. Statistical methods used in the Cluster-aware Multistage Selection to identify age-common and age-specific factors of *Plasmodium* infection among children under five in Nigeria, using the 2018 Nigeria Demographic and Health Survey

1. Weighted Least Absolute Shrinkage and Selection Operator

The least absolute shrinkage and selection operator (LASSO) performs simultaneous coefficient shrinkage and variable selection by imposing an L1 penalty that sets uninformative coefficients exactly to zero.[1,2] This is useful for Demographic and Health Survey (DHS) data, where many correlated individual- and community-level covariates can introduce instability. In Step 1 of our cluster-aware multistage selection (CMS), we applied survey-weighted LASSO to screen both main effects and age-interaction terms, retaining predictors most strongly associated with *Plasmodium* infection.

i) Model Specification

We modeled both age-group differences and within-group age trends using continuous age (6–59 months) and an indicator for ages 24–59 months:

$$\text{Plasmodium infection} \sim \text{age} + \text{age}_{24} + \sum_{k=1}^{N} X_k + \sum_{k=1}^{N} (\text{age}_{24}\colon X_k),$$

where X_k are the N covariates (N=56). Let X_i denote the full predictor vector (including interactions); the linear predictor is:

$$\eta_i = \beta_0 + \beta_{\text{age}}\,\text{age}_i + \beta_{\text{age24}}\,\text{age24}_i + \sum_{k=1}^{N} \beta_k X_{ik} + \sum_{k=1}^{N} \gamma_k\,(\text{age24}_i X_{ik})$$

Or simplified as;

$$\eta_i = X_i^{\top}\beta$$

and under this binomial model, predicted probability $\hat{\mu}_i = \Pr(Y_i = 1 \mid X_i)$.

ii) Penalized Objective

The weighted negative log-likelihood is:

$$L(\beta) = -\sum_{i=1}^{n} w_i[y_i \log \hat{\mu}_i + (1 - y_i)\log(1 - \hat{\mu}_i)],$$

and the LASSO adds an L1 penalty:

$$J(\beta) = L(\beta) + \lambda \sum_{j=1}^{m} \mid \beta_j \mid,$$

where λ (≥ 0) is the regularization parameter controlling shrinkage. Larger λ yields sparser models.

iii) Choosing λ with glmnet

We used the *glmnet* package (ver 4.1-10) in R to determine λ and run subsequent LASSO.[1] By default, *glmnet* evaluates a sequence of logarithmically spaced penalties $\{\lambda_1, \dots, \lambda_L\}$ between $\lambda_{\max}$ that shrinks all coefficients to zero (intercept-only model) and $\lambda_{\min} = 0.001 \times \lambda_{\max}$. For each λ, K-fold cross-validation computes the mean binomial deviance:

$$\mathrm{CV}(\lambda_l) = \frac{1}{K}\sum_{k=1}^{K} D^{(k)}(\lambda_l),$$

$$D^{(k)}(\lambda_l) = -2\sum_{i \in I_k} w_i[y_i \log \hat{\mu}_i(\lambda_l) + (1 - y_i)\log(1 - \hat{\mu}_i(\lambda_l))]$$

Two standard selection rules are used to determine λ:

- lambda.min – choose λ that minimizes CV error, allowing for more variable retention

- lambda.1se – choose the largest λ within 1 SE of the minimum, giving a more parsimonious model.

In our Step 1, the choice between these two rules was treated as a tuning dimension.

iv) glmnet LASSO for Binary Outcomes

glmnet does not directly penalize the logistic log-likelihood. Instead, it uses the standard iteratively reweighted least squares (IRLS) approximation, turning the logistic problem into a sequence of weighted Gaussian LASSO problems.

At IRLS iteration t the *glmnet* computes:

- Linear predictor: $\eta_i^{(t)} = X_i^\top \beta^{(t)}$
- Predicted probability: $\mu_i^{(t)} = \frac{1}{1+e^{-\eta_i^{(t)}}}$
- Working weight: $w_i^{(t)} = \mu_i^{(t)}(1 - \mu_i^{(t)})$
- Pseudo-outcome (working response): $z_i^{(t)} = \eta_i^{(t)} + \frac{y_i - \mu_i^{(t)}}{w_i^{(t)}}$

Then it defines the quadratic surrogate objective:

$$\min_{\beta} \ \frac{1}{2}\sum_i w_i^{(t)}[z_i^{(t)} - X_i^\top \beta]^2 + \lambda \sum_j \mid \beta_j \mid.$$

which corresponds to a Gaussian-style weighted least-squares approximation of the logistic likelihood. This surrogate is much easier to optimize, allowing *glmnet* to apply coordinate descent with soft-thresholding at every iteration.

v) Coordinate Descent and Soft-Thresholding

Within each IRLS iteration, *glmnet* updates coefficients one at a time:

$$\beta_j^{(t+1)} = \frac{\text{soft}\left(\sum_i w_i^{(t)} X_{ij}[z_i^{(t)} - X_{i,-j}^{\top}\beta_{-j}^{(t)}],\ \lambda\right)}{\sum_i w_i^{(t)} X_{ij}^2},$$

where

$$\text{soft}(u, \lambda) = \text{sign}(u)\ \max\left(\mid u \mid -\lambda, 0\right).$$

A coefficient is set to zero when:

$$\mid \sum_i w_i^{(t)} X_{ij}[z_i^{(t)} - X_{i,-j}^{\top}\beta_{-j}^{(t)}] \mid < \lambda,$$

meaning that the weighted association between X_j and the current residuals is insufficient to overcome the penalty. Practically, it means that the predictor contributes too little information to remain in the model. By selecting non-zero coefficient covariates through LASSO, we can screen our initial list of covariates.

2. *Stratified primary sampling unit-level bootstrapping*

To validate covariate selections that do not consider the stratification or clustering design of the Demographic and Health Survey (DHS) data, we bootstrapped our samples to assess stability of covariate selection. This involved resampling the dataset by their stratified primary sampling unit (PSU). We used 200 bootstrap replicates, as additional resampling beyond this threshold produced minimal improvement in selection stability relative to the computational cost. This was achieved by the following process.

Consider the bootstrap iterations $b = 1, \dots, 200$. For a clustered, stratified dataset with N number of participants, let S the total number of unique stratifications and H the total number of unique PSUs in the entire dataset. Let h_j be the number of unique PSUs within each stratum. Let n_{ij} be the number of individuals within each PSU h_j. This can be described as the following:

$$\sum_{j=1}^{S} h_j = H$$

$$\sum_{j=1}^{S} \sum_{i=1}^{j} n_{ij} = N$$

With stratified PSU-level bootstrapping, we resample *entire* PSUs within each stratum, preserving the DHS sampling structure. Specifically, if a stratum contains h_j PSUs, we draw n_{ij} PSUs with replacement from that same stratum. For example, if *Stratum 1* contains 4 PSUs and *Stratum 2* contains 5 PSUs, then each bootstrap replicate draws 4 PSUs (with replacement) from the Stratum 1 pool and 5 PSUs (with replacement) from the Stratum 2 pool. This means some PSUs may be selected multiple times and others not at all, exactly mirroring the logic of traditional bootstrap sampling while still respecting the DHS stratified design.

For each bootstrap replicate, we reran the covariate-selection procedure—specifically, Step 1 (survey-weighted LASSO) and Step 2 (generalized linear modeling to screen for candidate age-specific factors). We quantified the stability of each covariate by calculating its selection frequency across all bootstrap samples. Covariates that exceeded a prespecified selection-frequency threshold were retained for subsequent modeling.

1. Friedman J, Hastie T, Tibshirani R. Regularization paths for generalized linear models via coordinate descent. *J Stat Softw*. 2010;33(1). doi:10.18637/jss.v033.i01
2. Tibshirani R. Regression Shrinkage and Selection Via the Lasso. *Journal of the Royal Statistical Society Series B: Methodological*. 1996;58(1). doi:10.1111/j.2517-6161.1996.tb02080.x
