# Supplementary material for: Identifying age-common and age-specific factors of Plasmodium infection in Nigerian children under five: Application of a cluster-aware multistage selection framework to the 2018 Nigeria Demographic and Health Survey
Source: PLOS Glob Public Health. 2026 Jul 14;6(7):e0006693. doi: 10.1371/journal.pgph.0006693 (PMC13367690; doi:10.1371/journal.pgph.0006693)
Supplement: Attachment — Submitted filename: Response_to_Reviewers_auresp_2.docx [file pgph.0006693.s011.docx]

**Response to Reviewers**

We thank the Editor and Reviewers for their thoughtful and constructive feedback. We have carefully revised the manuscript to address all comments. Below, we provide a point-by-point response and indicate where changes have been made in the revised manuscript.

| Editor comment | Location | Response |
| --- | --- | --- |
| Please reformat the abstract as an unstructured abstract to comply with the journal’s requirements. | Abstract, page 3 | We reformatted the abstract into a fully unstructured format, removing section headers and presenting it as a continuous narrative. |
| Please proof-read the manuscript carefully. The text presently shifts between first-person and third-person pronouns; adopting one pronoun style consistently will improve clarity and coherence. | Throughout manuscript (notably Introduction and Methods) | We standardized the manuscript to consistently use first-person plural (“we”) throughout. |
| Please restructure the Discussion section by removing the subheadings and presenting the key findings in relation to existing literature, and highlighting the unique contributions of this study, where applicable. Additionally, the limitations could be presented as a single paragraph before the Conclusion section, without a separate heading. | Discussion section, pages 20-25 | We removed subheadings and reorganized the Discussion into a cohesive narrative. Limitations are now presented as a single paragraph before the Conclusion |
| Figure Resubmissions:  While revising your submission, we strongly recommend that you use PLOS’s NAAS tool (<https://ngplosjournals.pagemajik.ai/artanalysis>) to test your figure files. NAAS can convert your figure files to the TIFF file type and meet basic requirements (such as print size, resolution), or provide you with a report on issues that do not meet our requirements and that NAAS cannot fix. | Introduction section, page 8, Line 129, Figure 1  Results section, page 12, Line 210, Figure 2 | We have used the recommended tools to fix the tiff figure file and have pasted the modified figure for both figure 1 and figure 2. |

| Reviewer #1 comment | Location | Response |
| --- | --- | --- |
| The authors need to update presentation of results in the abstract section. The results are presented in a way that is confusing to someone who may be reading the paper the first time, and for someone who has reviewed this work an indication of 'laziness' to properly quote the PR and 95% CI for every statement result. | Abstract, page 3 | We reformatted the abstract into a fully unstructured format, removing section headers and presenting it as a continuous narrative. |
| The research is about Plasmodium infection in this population. In a number of sections the authors referred to this as malaria, please revise for consistency. | Throughout manuscript | We standardized the manuscript to consistently use first-person plural (“we”) throughout. |
| The analysis was done from data from 2018, almost a decade ago. Time may be an issue here. | Discussion section, page 24, Lines 396-399 | The more recent DHS (2024) has discontinued Plasmodium infection testing, and MIS (2021) do not collect covariate data as comprehensive as DHS. Therefore, the 2018 DHS was the most recent, comprehensive dataset that could best fulfill our aim of analyzing factors associated with Plasmodium infection. |
| The discussion section has improved a little, but better description on why PR is higher among households with ITNs, while PR is lower among children using ITNs. | Discussion section, page 22, Lines 353-362 | We expanded the Discussion to explain this using residual confounding, programmatic targeting, and access vs utilization differences. |

| Reviewer #2 comment | Location | Response |
| --- | --- | --- |
| The authors have carefully explained and justified responses to this Reviewer’s questions and concerns. The reason why children 5 years and older were not included in the analysis is now explained in the text. Revision of certain terms (e.g. Plasmodium infection) has been completed. They have done a nice job of developing hypotheses for what associations were predicted and why. The value of the conceptual framework (Fig. 1) is better explained, and is now presented as a Directed Acyclic Graph which is much more useful as a complement to the analytical plan. Interaction terms are now included as part of the analysis. The authors have briefly compared their findings with those from other studies, in particular by identifying the likely bidirectional feedback in some of the observed associations | NA | We thank the reviewer for their positive evaluation. All previously raised concerns have been addressed, and no further revisions were required. |
