# Supplementary material for: Identifying age-common and age-specific factors of Plasmodium infection in Nigerian children under five: Application of a cluster-aware multistage selection framework to the 2018 Nigeria Demographic and Health Survey
Source: PLOS Glob Public Health. 2026 Jul 14;6(7):e0006693. doi: 10.1371/journal.pgph.0006693 (PMC13367690; doi:10.1371/journal.pgph.0006693)
Supplement: Attachment — Submitted filename: Response to reviewers.docx [file pgph.0006693.s010.docx]

Reviewer #1:

-The authors mentioned the data was collected from a cross sectional study. In the abstract results section, presentation of important results was not clear. Proper presentation of results including OR and 95% CI should be included to guide the readers early. This should be done for the most important variables.

**Line 29-66**
Thank you for your comment. We have revised the abstract to clearly present the most important results, including point estimates and 95% confidence intervals, to guide readers early in the manuscript.

-Variable selection process from univariable to multivariable logistic regression using a cut-off p-value is not the most reliable method. Did the authors consider backward/forward stepwise regression approach?

**Line 137-202**
Thank you for your comment. Acknowledging that our prior approach may not have been sufficiently rigorous for population-level inference, we refined our analytic strategy using a multistage variable selection process. This approach does not rely solely on univariable p-values, but incorporates collinearity assessment through LASSO regression, followed by backward and forward selection using thresholds based on statistical significance, QIC, and confounding criteria.

-The use and interpretation of OR in a cross sectional study is not ideal. At best, a cross sectional study will tell us about prevalence of a disease thus the appropriate measure of association would be prevalence odds ratios (POR).

**Across manuscript**
We have modified our manuscript to present prevalence ratios rather than odds ratios and updated our statistical analysis accordingly, using appropriate regression methods for cross-sectional data.

-Conclusion section (lines 340-347) does not say much and is very vague. In public health studies of this nature, what is the overall message concerning modifiable risk factors that can result in lower prevalence of malaria in the age groups. Many of the independent variables analyzed in this study are not modifiable risk factors and thus do not provide any actionable information that would help policy or prevention strategies

despite significant association with malaria.

**Line 429-439**
Thank you for your comment. We have revised the conclusion to more clearly articulate the main contributions of our study: (1) identification of age-related heterogeneity within children under five that may inform prioritization of public health interventions, and (2) development of a reproducible cluster-aware multistage selection (CMS) framework applicable to DHS-like population-based surveys. We have also added suggestions for future public health interventions and studies to build upon our findings

-Limitation section (lines 335-336): authors state that this study does not have causal interpretation. Obviously from the statistical analysis methods used there is no possibility of causal interpretation, am not sure how this can be considered a limitation.

**Line 418-420**

We have revised the limitation section and removed statements implying causal interpretation.

-If possible please improve tabular presentation of results. It was not easy to read through and follow the results as currently presented.

**Table 1A, 1B, 2**

We have revised the tables to improve clarity, readability, and interpretability.

Reviewer #2:

Data were drawn from the 2018 Nigeria DHS, a large, cross-sectional, active surveillance

program that has produced abundant epidemiological risk factor data for many diverse diseases in LMICs throughout the world. The goal of this study was “(t)o better understand how malaria outcomes and associated factors may differ between younger and older children over the age of 2 (24-59 months) as this would encourage future interventions to target younger and older children differently for increased effectiveness. The authors were appropriately conservative in defining malaria “positive” status, by indicating: “To increase specificity, children who had both results of LM and RDT as negative were considered to not have malaria and those with only one negative result, but no result of the other testing method were excluded from analysis” (ln 88).

**Line 69-71, 99-102, 420-422**
Our analysis focused on children aged 6–59 months because laboratory-confirmed Plasmodium testing in the DHS is systematically conducted only among under-five children, who are also the population at highest risk for severe malaria outcomes. Although school-age children are increasingly recognized as an important reservoir of infection, they are not routinely tested in DHS surveys and therefore could not be included. We have clarified this rationale in the Introduction **(Line 69-71, Line 99-102)** and noted this as a limitation inherent to the DHS design **(Line 420-422).**

Malaria is not an infection, it is a disease. The authors should change “malaria infection” to “Plasmodium infection” throughout. The RDT or LM results, which indicate Plasmodium infection, may or may not demonstrate malaria disease, as many children (especially the older group) can be Plasmodium-positive without having malaria-like symptoms. This is especially true in an active, household-visit surveillance where the result of a blood test (but not of symptoms) is the only criterion used to designate each child’s status. Change “malaria status” to “infection status” or “Plasmodium status” throughout. This nuance of

infection vs. disease is especially important to consider when interpreting and discussing the results of the study.

**Across manuscript**
Thank you for this important clarification. We have revised the manuscript throughout to refer to Plasmodium infection rather than malaria, and adjusted terminology accordingly.

A conceptual framework for underlying risk factors is presented (Fig. 1), but no hypotheses are stated. What is the origin of this diagrammatic framework and how does it help in the design of questions and analysis that were used? Without such information, this research could be considered just a “fishing expedition.” What is the rationale for choosing “malaria” as the only “outcome” in the statistical models? Could “malaria” be an underlying cause of malnutrition or other factors that are “upstream” in the pathway diagram? If so, how might that change the analysis? Why are there no “return” arrows to indicate possible feedback, as has been demonstrated in many studies of malaria epidemiology? Why were interactions between variables not considered in the multivariable regressions? This might help disentangle risk factors for exposure to, and detection of, Plasmodium infection.

**Line 90-93, 114-115, 137-202**
Thank you for this thoughtful comment. We have added an explicit hypothesis at the end of the Introduction, positing that associations between risk factors and Plasmodium infection differ between younger (6–23.9 months) and older (24–59 months) under-five children due to differences in vulnerability and environmental exposure **(Line 90-93)**.

The conceptual framework was adapted from prior malaria epidemiology literature and used to guide covariate selection, rather than to imply a causal pathway. Plasmodium infection was selected as the outcome because DHS laboratory testing captures infection status rather than clinical disease, and our objective was to examine exposure-related risk patterns rather than downstream consequences. Although we acknowledge bidirectional relationships between infection and factors such as nutrition and socioeconomic status, feedback pathways were not depicted to maintain focus on exposure-associated factors **(Line 114-115)**

To address effect heterogeneity, we incorporated interaction terms between age group and selected covariates in the multivariable models; higher-order interactions among all covariates were not explored due to concerns about model complexity, collinearity, interpretability, and because such interactions were beyond the primary objective of the analysis **(Line 137-202)**

The authors should consider how they might include, or at least cite, the approach and figures presented in these articles:

de Castro and Fisher. (2012). Malaria Journal 11:161

http://www.malariajournal.com/content/11/1/161

Tusting et al. (2016). Infectious Diseases of Poverty 5:78

DOI 10.1186/s40249-016-0164-3

Jezek and Bamodu (2021) Health Res Policy Sys 19:93

https://doi.org/10.1186/s12961-021-00700-6

**Line 350-353**
We thank the reviewer for these suggested references and although we have not included the specific reference recommended, we have incorporated relevant conceptual insights including work by Tusting et al., into our discussion **(Line 350-353)**

In general, the Discussion needs to be developed further. While the authors recognize that this crosssectional study cannot determine causation, the suggestion that various risk factors “contribute to” malaria, should be considered more carefully. Anemia, poverty, rurality, agriculture, nutrition, mother’s knowledge, breastfeeding, ITN use (etc.) are all “correlated with” or “contribute to” Plasmodium infection and malaria disease, but this has already been shown in literally hundreds of studies. What is new here? One way to help

advance our understanding is to discuss the findings in the context of other studies that have shown similar, but also different, findings. Most of the associations that are discussed present only one citation that has also found the same association. There are many published reports on the epidemiology of Plasmodium infection and malaria disease in Nigeria that should be cited to introduce and justify this report. Similarly, comparing the results of the current report with those from other studies in the region would strengthen the value and validity of findings.

**Overall Discussion section, specifically Line 401-415**
We have substantially revised the Discussion to clarify the novel contributions of this study, including identification of age-specific heterogeneity within children under five, reconciliation of mixed findings in prior literature, and application of a rigorous, survey-aligned CMS framework incorporating interaction terms, complex survey design, and multistage variable selection. **(Line 401-415)**

A brief discussion of how the patterns for these younger children are similar to or different than those of other age groups could be informative. Even if not fully analyzed, briefly comparing infection patterns in older children and adults, as summarized in the 2018 Nigeria Demographic and Health Survey published report, would be helpful. In addition, a more thoughtful and considered comparison of this study’s major themes with those of other comparable studies would strengthen this report. Similarly, an analysis that considers how positive and negative “feedback” (as mentioned previously) might affect the associations, even producing counter-intuitive results, would enlighten readers.

**Line 376-395, 350-355, 356-360, 362-365**
We have added a brief descriptive comparison in the Discussion, comparing our findings with older age studies and suggesting possible mechanistic similarities **(Line 376-395)**. We have also discussed about potential bidirectional associations or counter-intuitive associations (ex. ITN use associated with high prevalence) between malaria and the variables we have assessed **(Line 350-355, 356-360, 362-365)**.

Anopheles mosquitoes are not simply the “primary vector” of Plasmodium spp. to humans; certain Anopheles species are the sole vectors. It is naïve to treat all vector-competent Anopheles species as the same. e.g. (ln. 278) where it is stated that “This exposes the children to vector-friendly environments as agricultural land harbors water pools without nearby vegetation.” A few important Anopheles species almost exclusively breed in water with vegetation, or in human-made water containers. The discussion of Anopheles vectors should be corrected.

**Line 337-349**
We have revised the discussion of Anopheles vectors to reflect species-specific ecological heterogeneity and now rely on prior geospatial analyses rather than simplified generalizations. **(Line 337-349)**

Ln 86: Define LM and RDT on first use

**Line 106-108**

LM was not defined as it was not used after initial use.

Ln 132: Remove the three stippled bars in Fig. 2 (they add no information)

**Figure 2**

The strippled bars were removed and only the positive Plasmodium data were presented

Ln 310: Remove the word “effect” in “… differential effect of educational factors …”

The sentence was replaced completely

Ln 341: Change the word “impacts” in “ … analyze the differential impacts of …”

6.

The sentence was replaced completely
