# Supplementary material for: Identifying age-common and age-specific factors of Plasmodium infection in Nigerian children under five: Application of a cluster-aware multistage selection framework to the 2018 Nigeria Demographic and Health Survey
Source: PLOS Glob Public Health. 2026 Jul 14;6(7):e0006693. doi: 10.1371/journal.pgph.0006693 (PMC13367690; doi:10.1371/journal.pgph.0006693)
Supplement: Peer review history

# Peer review: Decision Letter 0

**DOI:** 10.1371/journal.pgph.0006693.r001

**Authors:** Rajiv Sarkar

2 Aug 2025

PGPH-D-25-01049

Age-specific factors associated with Malaria among children of age 6-23.9 months and 24-59 months in Nigeria using the 2018 Demographic Health and Survey data.

PLOS Global Public Health

Dear Dr. Choi,

Thank you for submitting your manuscript to PLOS Global Public Health. After careful consideration, we feel that it has merit but does not fully meet PLOS Global Public Health’s publication criteria as it currently stands. Therefore, we invite you to submit a revised version of the manuscript that addresses the points raised during the review process.

Please submit your revised manuscript by Sep 16 2025 11:59PM. If you will need more time than this to complete your revisions, please reply to this message or contact the journal office at globalpubhealth@plos.org. When you're ready to submit your revision, log on to https://www.editorialmanager.com/pgph/ and select the 'Submissions Needing Revision' folder to locate your manuscript file.

Please include the following items when submitting your revised manuscript:

- A rebuttal letter that responds to each point raised by the editor and reviewer(s). You should upload this letter as a separate file labeled 'Response to Reviewers'.
- A marked-up copy of your manuscript that highlights changes made to the original version. You should upload this as a separate file labeled 'Revised Manuscript with Track Changes'.
- An unmarked version of your revised paper without tracked changes. You should upload this as a separate file labeled 'Manuscript'.

Guidelines for resubmitting your figure files are available below the reviewer comments at the end of this letter.

We look forward to receiving your revised manuscript.

Kind regards,

Rajiv Sarkar

Academic Editor

PLOS Global Public Health

Journal Requirements:

1. We have amended your Competing Interest statement to comply with journal style. We kindly ask that you double check the statement and let us know if anything is incorrect.

2. Please provide a/amend your detailed Financial Disclosure statement. This is published with the article. It must therefore be completed in full sentences and contain the exact wording you wish to be published.

If you did not receive any funding for this study, please simply state: “The authors received no specific funding for this work.”

3. In the online submission form, you indicated that Data described in the manuscript, code book, and analytic code are publicly available in the DHS website by request.

All PLOS journals now require all data underlying the findings described in their manuscript to be freely available to other researchers, either

1. In a public repository,

2. Within the manuscript itself, or

3. Uploaded as supplementary information.

This policy applies to all data except where public deposition would breach compliance with the protocol approved by your research ethics board. If your data cannot be made publicly available for ethical or legal reasons (e.g., public availability would compromise patient privacy), please explain your reasons by return email and your exemption request will be escalated to the editor for approval. Your exemption request will be handled independently and will not hold up the peer review process, but will need to be resolved should your manuscript be accepted for publication. One of the Editorial team will then be in touch if there are any issues.

4. Please provide separate figure files in .tif or .eps format.

For more information about figure files please see our guidelines:

https://journals.plos.org/globalpublichealth/s/figures

https://journals.plos.org/globalpublichealth/s/figures#loc-file-requirements

5. Please upload a copy of Figure 1, 2, 3 which you refer to in your text on page 6, 8, 16. Or, if the figure is no longer to be included as part of the submission please remove all reference to it within the text.

If the reviewer comments include a recommendation to cite specific previously published works, please review and evaluate these publications to determine whether they are relevant and should be cited. There is no requirement to cite these works unless the editor has indicated otherwise.

[Note: HTML markup is below. Please do not edit.]

Reviewers' comments:

Reviewer's Responses to Questions

**Comments to the Author**

1. Does this manuscript meet PLOS Global Public Health’s publication criteria? Is the manuscript technically sound, and do the data support the conclusions? The manuscript must describe methodologically and ethically rigorous research with conclusions that are appropriately drawn based on the data presented.

Reviewer #1: Partly

Reviewer #2: Partly

**********

2. Has the statistical analysis been performed appropriately and rigorously?

Reviewer #1: No

Reviewer #2: Yes

**********

3. Have the authors made all data underlying the findings in their manuscript fully available (please refer to the Data Availability Statement at the start of the manuscript PDF file)?

The PLOS Data policy requires authors to make all data underlying the findings described in their manuscript fully available without restriction, with rare exception. The data should be provided as part of the manuscript or its supporting information, or deposited to a public repository. For example, in addition to summary statistics, the data points behind means, medians and variance measures should be available. If there are restrictions on publicly sharing data—e.g. participant privacy or use of data from a third party—those must be specified.

Reviewer #1: Yes

Reviewer #2: Yes

**********

4. Is the manuscript presented in an intelligible fashion and written in standard English?

PLOS Global Public Health does not copyedit accepted manuscripts, so the language in submitted articles must be clear, correct, and unambiguous. Any typographical or grammatical errors should be corrected at revision, so please note any specific errors here.

Reviewer #1: Yes

Reviewer #2: Yes

**********

5. Review Comments to the Author

Please use the space provided to explain your answers to the questions above. You may also include additional comments for the author, including concerns about dual publication, research ethics, or publication ethics. (Please upload your review as an attachment if it exceeds 20,000 characters)

Reviewer #1: -The authors mentioned the data was collected from a cross sectional study. In the abstract results section, presentation of important results was not clear. Proper presentation of results including OR and 95% CI should be included to guide the readers early. This should be done for the most important variables.

-Variable selection process from univariable to multivariable logistic regression using a cut-off p-value is not the most reliable method. Did the authors consider backward/forward stepwise regression approach?

-The use and interpretation of OR in a cross sectional study is not ideal. At best, a cross sectional study will tell us about prevalence of a disease thus the appropriate measure of association would be prevalence odds ratios (POR).

-Conclusion section (lines 340-347) does not say much and is very vague. In public health studies of this nature, what is the overall message concerning modifiable risk factors that can result in lower prevalence of malaria in the age groups. Many of the independent variables analyzed in this study are not modifiable risk factors and thus do not provide any actionable information that would help policy or prevention strategies despite significant association with malaria.

-Limitation section (lines 335-336): authors state that this study does not have causal interpretation. Obviously from the statistical analysis methods used there is no possibility of causal interpretation, am not sure how this can be considered a limitation.

-If possible please improve tabular presentation of results. It was not easy to read through and follow the results as currently presented.

Reviewer #2: Data were drawn from the 2018 Nigeria DHS, a large, cross-sectional, active surveillance program that has produced abundant epidemiological risk factor data for many diverse diseases in LMICs throughout the world. The goal of this study was “(t)o better understand how malaria outcomes and associated factors may differ between younger and older children over the age of 2 (24-59 months) as this would encourage future interventions to target younger and older children differently for increased effectiveness. The authors were appropriately conservative in defining malaria “positive” status, by indicating: “To increase specificity, children who had both results of LM and RDT as negative were considered to not have malaria and those with only one negative result, but no result of the other testing method were excluded from analysis” (ln 88).

Major Issues

The data from children under five were analyzed, but older children (i.e. “school-age children”) were not considered. The authors should better explain why only children aged less than 5 yrs were chosen, and why older children were not included in the analysis. This is particularly important because of increasing evidence that “school-age children” are very often infected with Plasmodium, but are much less often ill with malaria. Whether under-5 children need to be particularly targeted for malaria prevention might depend on the infection prevalence and/or disease burden in older children, as well as adults.

Malaria is not an infection, it is a disease. The authors should change “malaria infection” to “Plasmodium infection” throughout. The RDT or LM results, which indicate Plasmodium infection, may or may not demonstrate malaria disease, as many children (especially the older group) can be Plasmodium-positive without having malaria-like symptoms. This is especially true in an active, household-visit surveillance where the result of a blood test (but not of symptoms) is the only criterion used to designate each child’s status. Change “malaria status” to “infection status” or “Plasmodium status” throughout. This nuance of infection vs. disease is especially important to consider when interpreting and discussing the results of the study.

A conceptual framework for underlying risk factors is presented (Fig. 1), but no hypotheses are stated. What is the origin of this diagrammatic framework and how does it help in the design of questions and analysis that were used? Without such information, this research could be considered just a “fishing expedition.” What is the rationale for choosing “malaria” as the only “outcome” in the statistical models? Could “malaria” be an underlying cause of malnutrition or other factors that are “upstream” in the pathway diagram? If so, how might that change the analysis? Why are there no “return” arrows to indicate possible feedback, as has been demonstrated in many studies of malaria epidemiology? Why were interactions between variables not considered in the multivariable regressions? This might help disentangle risk factors for exposure to, and detection of, Plasmodium infection.

The authors should consider how they might include, or at least cite, the approach and figures presented in these articles:

de Castro and Fisher. (2012). Malaria Journal 11:161

http://www.malariajournal.com/content/11/1/161

Tusting et al. (2016). Infectious Diseases of Poverty 5:78

DOI 10.1186/s40249-016-0164-3

Jezek and Bamodu (2021) Health Res Policy Sys 19:93

https://doi.org/10.1186/s12961-021-00700-6

In general, the Discussion needs to be developed further. While the authors recognize that this cross-sectional study cannot determine causation, the suggestion that various risk factors “contribute to” malaria, should be considered more carefully. Anemia, poverty, rurality, agriculture, nutrition, mother’s knowledge, breastfeeding, ITN use (etc.) are all “correlated with” or “contribute to” Plasmodium infection and malaria disease, but this has already been shown in literally hundreds of studies. What is new here? One way to help advance our understanding is to discuss the findings in the context of other studies that have shown similar, but also different, findings. Most of the associations that are discussed present only one citation that has also found the same association. There are many published reports on the epidemiology of Plasmodium infection and malaria disease in Nigeria that should be cited to introduce and justify this report. Similarly, comparing the results of the current report with those from other studies in the region would strengthen the value and validity of findings.

A brief discussion of how the patterns for these younger children are similar to or different than those of other age groups could be informative. Even if not fully analyzed, briefly comparing infection patterns in older children and adults, as summarized in the 2018 Nigeria Demographic and Health Survey published report, would be helpful. In addition, a more thoughtful and considered comparison of this study’s major themes with those of other comparable studies would strengthen this report. Similarly, an analysis that considers how positive and negative “feedback” (as mentioned previously) might affect the associations, even producing counter-intuitive results, would enlighten readers.

Anopheles mosquitoes are not simply the “primary vector” of Plasmodium spp. to humans; certain Anopheles species are the sole vectors. It is naïve to treat all vector-competent Anopheles species as the same. e.g. (ln. 278) where it is stated that “This exposes the children to vector-friendly environments as agricultural land harbors water pools without nearby vegetation.” A few important Anopheles species almost exclusively breed in water with vegetation, or in human-made water containers. The discussion of Anopheles vectors should be corrected.

Minor

Ln 86: Define LM and RDT on first use

Ln 132: Remove the three stippled bars in Fig. 2 (they add no information)

Ln 310: Remove the word “effect” in “… differential effect of educational factors …”

Ln 341: Change the word “impacts” in “ … analyze the differential impacts of …”

**********

6. PLOS authors have the option to publish the peer review history of their article (what does this mean?). If published, this will include your full peer review and any attached files.

**Do you want your identity to be public for this peer review?**  If you choose “no”, your identity will remain anonymous but your review may still be made public.

For information about this choice, including consent withdrawal, please see our Privacy Policy.

Reviewer #1: No

Reviewer #2: No

**********

[NOTE: If reviewer comments were submitted as an attachment file, they will be attached to this email and accessible via the submission site. Please log into your account, locate the manuscript record, and check for the action link "View Attachments". If this link does not appear, there are no attachment files.]

While revising your submission, please upload your figure files to the Preflight Analysis and Conversion Engine (PACE) digital diagnostic tool, https://pacev2.apexcovantage.com/. PACE helps ensure that figures meet PLOS requirements. To use PACE, you must first register as a user. Registration is free. Then, login and navigate to the UPLOAD tab, where you will find detailed instructions on how to use the tool. If you encounter any issues or have any questions when using PACE, please email PLOS at figures@plos.org. Please note that Supporting Information files do not need this step.

# Author comment: Author response to Decision Letter 1

**DOI:** 10.1371/journal.pgph.0006693.r002

30 Dec 2025

Attachment Submitted filename: Response to reviewers.docx

# Peer review: Decision Letter 1

**DOI:** 10.1371/journal.pgph.0006693.r003

**Authors:** Rajiv Sarkar, Rajiv Sarkar

12 Feb 2026

PGPH-D-25-01049R1

Identifying age-common and age-specific factors of Plasmodium infection in Nigerian children under five: application of a cluster-aware multistage selection framework to the 2018 Nigeria Demographic and Health Survey

PLOS Global Public Health

Dear Dr. Kang,

Thank you for submitting your manuscript to PLOS Global Public Health. After careful consideration, we feel that it has merit but does not fully meet PLOS Global Public Health’s publication criteria as it currently stands. Therefore, we invite you to submit a revised version of the manuscript that addresses the points raised during the review process.

Please submit your revised manuscript by Mar 14 2026 11:59PM. If you will need more time than this to complete your revisions, please reply to this message or contact the journal office at globalpubhealth@plos.org. When you're ready to submit your revision, log on to https://www.editorialmanager.com/pgph/ and select the 'Submissions Needing Revision' folder to locate your manuscript file.

Please include the following items when submitting your revised manuscript:

- A letter that responds to each point raised by the editor and reviewer(s). You should upload this letter as a separate file labeled 'Response to Reviewers'.
- A marked-up copy of your manuscript that highlights changes made to the original version. You should upload this as a separate file labeled 'Revised Manuscript with Track Changes'.
- An unmarked version of your revised paper without tracked changes. You should upload this as a separate file labeled 'Manuscript'.

Guidelines for resubmitting your figure files are available below the reviewer comments at the end of this letter.

We look forward to receiving your revised manuscript.

Kind regards,

Rajiv Sarkar

Academic Editor

PLOS Global Public Health

Journal Requirements:

If the reviewer comments include a recommendation to cite specific previously published works, please review and evaluate these publications to determine whether they are relevant and should be cited. There is no requirement to cite these works unless the editor has indicated otherwise.

Please review your reference list to ensure that it is complete and correct. If you have cited papers that have been retracted, please include the rationale for doing so in the manuscript text, or remove these references and replace them with relevant current references. Any changes to the reference list should be mentioned in the rebuttal letter that accompanies your revised manuscript. If you need to cite a retracted article, indicate the article’s retracted status in the References list and also include a citation and full reference for the retraction notice.

Additional Editor Comments (if provided):

1. Please reformat the abstract as an unstructured abstract to comply with the journal’s requirements.

2. Please proof-read the manuscript carefully. The text presently shifts between first-person and third-person pronouns; adopting one pronoun style consistently will improve clarity and coherence.

3. Please restructure the Discussion section by removing the subheadings and presenting the key findings in relation to existing literature, and highlighting the unique contributions of this study, where applicable. Additionally, the limitations could be presented as a single paragraph before the Conclusion section, without a separate heading.

Reviewers' comments:

Reviewer's Responses to Questions

**Comments to the Author**

1. If the authors have adequately addressed your comments raised in a previous round of review and you feel that this manuscript is now acceptable for publication, you may indicate that here to bypass the “Comments to the Author” section, enter your conflict of interest statement in the “Confidential to Editor” section, and submit your "Accept" recommendation.

Reviewer #1: (No Response)

Reviewer #2: All comments have been addressed

**********

2. Does this manuscript meet PLOS Global Public Health’s publication criteria? Is the manuscript technically sound, and do the data support the conclusions? The manuscript must describe methodologically and ethically rigorous research with conclusions that are appropriately drawn based on the data presented.

Reviewer #1: Partly

Reviewer #2: Yes

**********

3. Has the statistical analysis been performed appropriately and rigorously?

Reviewer #1: Yes

Reviewer #2: Yes

**********

4. Have the authors made all data underlying the findings in their manuscript fully available (please refer to the Data Availability Statement at the start of the manuscript PDF file)?

The PLOS Data policy requires authors to make all data underlying the findings described in their manuscript fully available without restriction, with rare exception. The data should be provided as part of the manuscript or its supporting information, or deposited to a public repository. For example, in addition to summary statistics, the data points behind means, medians and variance measures should be available. If there are restrictions on publicly sharing data—e.g. participant privacy or use of data from a third party—those must be specified.

Reviewer #1: Yes

Reviewer #2: Yes

**********

5. Is the manuscript presented in an intelligible fashion and written in standard English?

PLOS Global Public Health does not copyedit accepted manuscripts, so the language in submitted articles must be clear, correct, and unambiguous. Any typographical or grammatical errors should be corrected at revision, so please note any specific errors here.

Reviewer #1: Yes

Reviewer #2: Yes

**********

6. Review Comments to the Author

Please use the space provided to explain your answers to the questions above. You may also include additional comments for the author, including concerns about dual publication, research ethics, or publication ethics. (Please upload your review as an attachment if it exceeds 20,000 characters)

Reviewer #1: The authors need to update presentation of results in the abstract section. The results are presented in a way that is confusing to someone who may be reading the paper the first time, and for someone who has reviewed this work an indication of 'laziness' to properly quote the PR and 95% CI for every statement result.

The research is about Plasmodium infection in this population. In a number of sections the authors referred to this as malaria, please revise for consistency.

The analysis was done from data from 2018, almost a decade ago. Time may be an issue here.

The discussion section has improved a little, but better description on why PR is higher among households with ITNs, while PR is lower among children using ITNs.

Reviewer #2: The authors have carefully explained and justified responses to this Reviewer’s questions and concerns. The reason why children 5 years and older were not included in the analysis is now explained in the text. Revision of certain terms (e.g. Plasmodium infection) has been completed. They have done a nice job of developing hypotheses for what associations were predicted and why. The value of the conceptual framework (Fig. 1) is better explained, and is now presented as a Directed Acyclic Graph which is much more useful as a complement to the analytical plan. Interaction terms are now included as part of the analysis. The authors have briefly compared their findings with those from other studies, in particular by identifying the likely bidirectional feedback in some of the observed associations.

**********

7. PLOS authors have the option to publish the peer review history of their article (what does this mean?). If published, this will include your full peer review and any attached files.

**Do you want your identity to be public for this peer review?**  If you choose “no”, your identity will remain anonymous but your review may still be made public.

For information about this choice, including consent withdrawal, please see our Privacy Policy.

Reviewer #1: No

Reviewer #2: No

**********

[NOTE: If reviewer comments were submitted as an attachment file, they will be attached to this email and accessible via the submission site. Please log into your account, locate the manuscript record, and check for the action link "View Attachments". If this link does not appear, there are no attachment files.]

Figure Resubmissions:

While revising your submission, we strongly recommend that you use PLOS’s NAAS tool (https://ngplosjournals.pagemajik.ai/artanalysis) to test your figure files. NAAS can convert your figure files to the TIFF file type and meet basic requirements (such as print size, resolution), or provide you with a report on issues that do not meet our requirements and that NAAS cannot fix.

After uploading your figures to PLOS’s NAAS tool - https://ngplosjournals.pagemajik.ai/artanalysis, NAAS will process the files provided and display the results in the "Uploaded Files" section of the page as the processing is complete. If the uploaded figures meet our requirements (or NAAS is able to fix the files to meet our requirements), the figure will be marked as "fixed" above. If NAAS is unable to fix the files, a red "failed" label will appear above. When NAAS has confirmed that the figure files meet our requirements, please download the file via the download option, and include these NAAS processed figure files when submitting your revised manuscript.

# Author comment: Author response to Decision Letter 2

**DOI:** 10.1371/journal.pgph.0006693.r004

7 Apr 2026

Attachment Submitted filename: Response_to_Reviewers_auresp_2.docx

# Peer review: Decision Letter 2

**DOI:** 10.1371/journal.pgph.0006693.r005

**Authors:** Rajiv Sarkar, Rajiv Sarkar, Julia Robinson

2 Jun 2026

Identifying age-common and age-specific factors of Plasmodium infection in Nigerian children under five: application of a cluster-aware multistage selection framework to the 2018 Nigeria Demographic and Health Survey

PGPH-D-25-01049R2

Dear Dr Kang,

We are pleased to inform you that your manuscript 'Identifying age-common and age-specific factors of Plasmodium infection in Nigerian children under five: application of a cluster-aware multistage selection framework to the 2018 Nigeria Demographic and Health Survey' has been provisionally accepted for publication in PLOS Global Public Health.

Before your manuscript can be formally accepted you will need to complete some formatting changes, which you will receive in a follow up email. A member of our team will be in touch with a set of requests.

Please note that your manuscript will not be scheduled for publication until you have made the required changes, so a swift response is appreciated.

IMPORTANT: The editorial review process is now complete. PLOS will only permit corrections to spelling, formatting or significant scientific errors from this point onwards. Requests for major changes, or any which affect the scientific understanding of your work, will cause delays to the publication date of your manuscript.

If your institution or institutions have a press office, please notify them about your upcoming paper to help maximize its impact. If they'll be preparing press materials, please inform our press team as soon as possible -- no later than 48 hours after receiving the formal acceptance. Your manuscript will remain under strict press embargo until 2 pm Eastern Time on the date of publication. For more information, please contact globalpubhealth@plos.org.

Thank you again for supporting Open Access publishing; we are looking forward to publishing your work in PLOS Global Public Health.

Best regards,

Julia Robinson

Executive Editor

PLOS Global Public Health

***********************************************************

Reviewer Comments (if any, and for reference):

Reviewer's Responses to Questions

**Comments to the Author**

1. If the authors have adequately addressed your comments raised in a previous round of review and you feel that this manuscript is now acceptable for publication, you may indicate that here to bypass the “Comments to the Author” section, enter your conflict of interest statement in the “Confidential to Editor” section, and submit your "Accept" recommendation.

Reviewer #2: All comments have been addressed

**********

2. Does this manuscript meet PLOS Global Public Health’s publication criteria? Is the manuscript technically sound, and do the data support the conclusions? The manuscript must describe methodologically and ethically rigorous research with conclusions that are appropriately drawn based on the data presented.

Reviewer #2: (No Response)

**********

3. Has the statistical analysis been performed appropriately and rigorously?

Reviewer #2: (No Response)

**********

4. Have the authors made all data underlying the findings in their manuscript fully available (please refer to the Data Availability Statement at the start of the manuscript PDF file)?

The PLOS Data policy requires authors to make all data underlying the findings described in their manuscript fully available without restriction, with rare exception. The data should be provided as part of the manuscript or its supporting information, or deposited to a public repository. For example, in addition to summary statistics, the data points behind means, medians and variance measures should be available. If there are restrictions on publicly sharing data—e.g. participant privacy or use of data from a third party—those must be specified.

Reviewer #2: (No Response)

**********

5. Is the manuscript presented in an intelligible fashion and written in standard English?

PLOS Global Public Health does not copyedit accepted manuscripts, so the language in submitted articles must be clear, correct, and unambiguous. Any typographical or grammatical errors should be corrected at revision, so please note any specific errors here.

Reviewer #2: (No Response)

**********

6. Review Comments to the Author

Please use the space provided to explain your answers to the questions above. You may also include additional comments for the author, including concerns about dual publication, research ethics, or publication ethics. (Please upload your review as an attachment if it exceeds 20,000 characters)

Reviewer #2: (No Response)

**********

7. PLOS authors have the option to publish the peer review history of their article (what does this mean?). If published, this will include your full peer review and any attached files.

**Do you want your identity to be public for this peer review?**  If you choose “no”, your identity will remain anonymous but your review may still be made public.

For information about this choice, including consent withdrawal, please see our Privacy Policy.

Reviewer #2: No

**********
